# Neural Substrates Related to Motor Memory with Multiple Timescales in Sensorimotor Adaptation

**DOI:** 10.1371/journal.pbio.1002312

**Published:** 2015-12-08

**Authors:** Sungshin Kim, Kenji Ogawa, Jinchi Lv, Nicolas Schweighofer, Hiroshi Imamizu

**Affiliations:** 1 Neuroscience Graduate Program, University of Southern California, Los Angeles, California, United States of America; 2 Feinberg School of Medicine, Northwestern University, Chicago, Illinois, United States of America; 3 Cognitive Mechanisms Laboratories, Advanced Telecommunications Research Institute International, Keihanna Science City, Kyoto, Japan; 4 Department of Psychology, Graduate School of Letters, Hokkaido University, Sapporo, Japan; 5 Data Sciences and Operations Department, Marshall School of Business, University of Southern California, Los Angeles, California, United States of America; 6 Division of Biokinesiology and Physical Therapy, University of Southern California, Los Angeles, California, United States of America; 7 EuroMov, Movement to Health Laboratory (M2H), Université Montpellier-1, Montpellier, France; 8 Center for Information and Neural Networks, National Institute of Information and Communications Technology and Osaka University, Suita, Osaka, Japan; 9 Department of Psychology, Graduate School of Humanities and Sociology, The University of Tokyo, Tokyo, Japan; University of Minnesota, UNITED STATES

## Abstract

Recent computational and behavioral studies suggest that motor adaptation results from the update of multiple memories with different timescales. Here, we designed a model-based functional magnetic resonance imaging (fMRI) experiment in which subjects adapted to two opposing visuomotor rotations. A computational model of motor adaptation with multiple memories was fitted to the behavioral data to generate time-varying regressors of brain activity. We identified regional specificity to timescales: in particular, the activity in the inferior parietal region and in the anterior-medial cerebellum was associated with memories for intermediate and long timescales, respectively. A sparse singular value decomposition analysis of variability in specificities to timescales over the brain identified four components, two fast, one middle, and one slow, each associated with different brain networks. Finally, a multivariate decoding analysis showed that activity patterns in the anterior-medial cerebellum progressively represented the two rotations. Our results support the existence of brain regions associated with multiple timescales in adaptation and a role of the cerebellum in storing multiple internal models.

## Abstract 2

A model-based functional MRI study reveals the existence of brain regions associated with four distinct timescales in motor adaptation.

## Author Summary

Motor adaptation, a form of motor learning in which motor commands are modified to compensate for disturbances in the external environment, usually proceeds at a rapid pace initially and is then followed by more gradual adjustments. This suggests that at least two learning processes are involved, but little is known about how many distinct memories the brain actually updates during motor adaptation. In addition, it is unclear whether these putative multiple motor memories reside within a single neural system that encompasses different timescales or in qualitatively distinct neural systems. We addressed these issues using a model-based functional imaging approach in which we first used behavioral data to derive a large number of possible memory “states,” each with different dynamics, and then correlated these memory states with neural activities. We identified four components: two fast, one intermediate, and one slow, each associated with different brain networks. In particular, areas in the prefrontal and parietal lobes and the posterior part of the cerebellum were associated with formation of memories for short timescales. By contrast, the inferior parietal region and the anterior-medial cerebellum were associated with formation of memories for intermediate and long timescales, respectively.

## Introduction

Behavioral and computational modeling studies, on the one hand, and neuroimaging studies, on the other hand, have greatly advanced our understanding of motor adaptation. In particular, recent behavioral and computational modeling studies have shed light on the temporal structure of motor adaptation by showing that motor behavior is well accounted for by the sum of multiple motor memory states with different timescales. For instance, models with two time constants can reproduce a number of adaptation phenomena such as anterograde interference, spontaneous recovery, and savings [1–4]. A model with a larger number of time constants can account for adaptation occurring at multiple timescales, e.g., fatigue and aging [5]. In contrast, neuroimaging studies, using either functional magnetic resonance imaging (fMRI) [6–8] or positron emission tomography (PET) [9–12], have investigated the spatial distribution of the neural correlates and plastic changes across different brain regions at specific times during and after adaptation, with the prefrontal cortex (PFC), the posterior parietal cortex (PPC), and the cerebellum consistently showing activation. The PFC mostly contributes to the early, but not the late, stage of adaptation, which is consistent with its role in spatial working memory and in attention and arousal at the onsets of target presentation [13,14]. The PPC is also important in the early stage of motor adaptation [6,9,15], here again consistent with its role in working memory [13,14], planning movements and early adaptation to a new visuomotor transformation [9,16,17]. The activity of the cerebellum increases in a later stage of visuomotor adaptation [6,15,18] and correlates with the degree of savings at transfer of learning [19]. Such activation is consistent with cerebellum learning from errors [20,21], building internal models [22–24], and storing multiple motor skills [25].

However, these modeling and neuroimaging studies have been conducted independently of each other. As a result, little is known about the neural correlates of the latent (i.e., nondirectly observable from the behavioral data) motor memories at different timescales suggested by computational models. In particular, it is unclear whether the multiple motor memories proposed by the models reside within a single system that contains a distribution of possible timescales or in a finite set of qualitatively distinguishable neural systems [1,26]. In addition, because experimental behavioral data can be well accounted for by models with different number of time constants, it is unclear how many distinct memories the brain actually updates during a specific type of motor adaptation. Finally, it is unclear whether the neural substrates identified in the early and late phases of adaptation in previous fMRI studies map onto putative “fast” and “slow” processes suggested by the computational models.

Here, we combined modeling and imaging approaches via a model-based fMRI study of the spatial and temporal distribution of multiple motor memories during adaptation. Subjects adapted to two opposing visuomotor rotations in short alternating blocks. We estimated the multiple memories via a multiple-timescale adaptation model that generalizes the two-state models to multiple states with a logarithmic distribution of time scales from seconds to hours, as in a previous study [5]. A model-based approach based on regression analyses of brain activity would have insufficient power to dissociate multiple memories that are highly correlated with each other. We therefore propose a novel two-step approach. In a first step, we conducted exploratory multiple single regressions with individual memories, which avoids the problem of multicollinearity. In a second step, we performed a sparse singular value decomposition (SVD) on the voxels identified in the first step, in order to select a small number of orthogonal components. As a result, we identified four characteristic networks, each associated with formation of different time-scales of memories.

## Results

**Fig 1:**
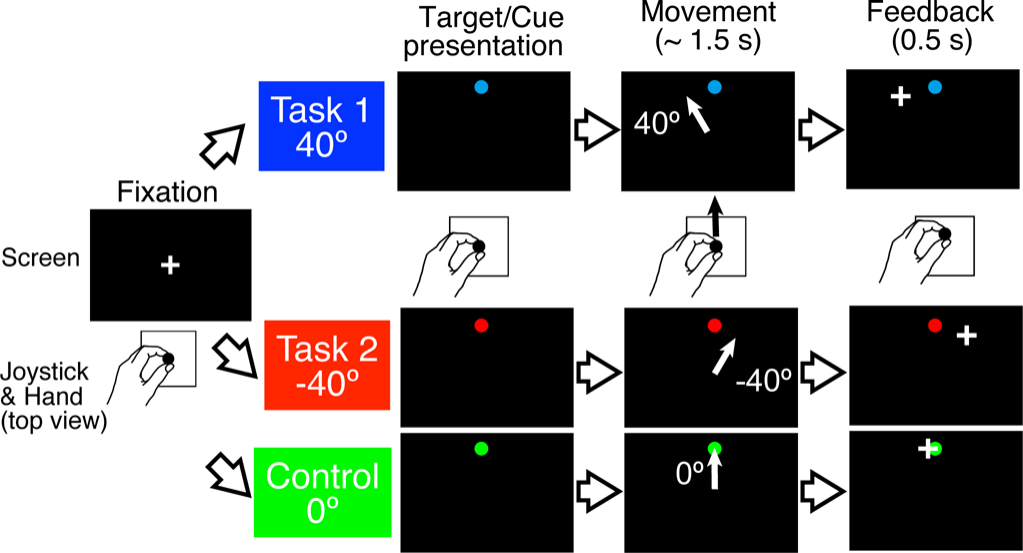
Single trial timeline. The cursor was rotated by 40° or −40° in adaptation trials or by 0° in control trials. A target signaled the start of the movement, which had to be completed within 1.5 s (maximum movement time) for the trial to be valid. Feedback was provided by showing a cursor position for 0.5 s after the maximum movement time. The different tasks were cued by target colors (blue, red, and green; see Materials and Methods).

Twenty-one healthy right-handed subjects used their left hand and adapted to two opposing visuomotor rotations, of 40° and −40°, respectively, presented in blocks of nine trials. At each trial, subjects were instructed to hit a circular target that appeared on the visual display by manipulating a joystick with their left hand, with the goal to decrease the distance between the cursor and the target at the end of the movement (see Fig 1 and details in Materials and Methods). The cursor was rotated 40°, −40°, or 0° from the actual movement direction depending on the task (Task 1: 40°; Task 2: −40°; Control task: 0°), which was cued by the target color (Fig 1). The tasks were presented in blocks of nine trials.

### Behavioral Results and Modeling

**Fig 2:**
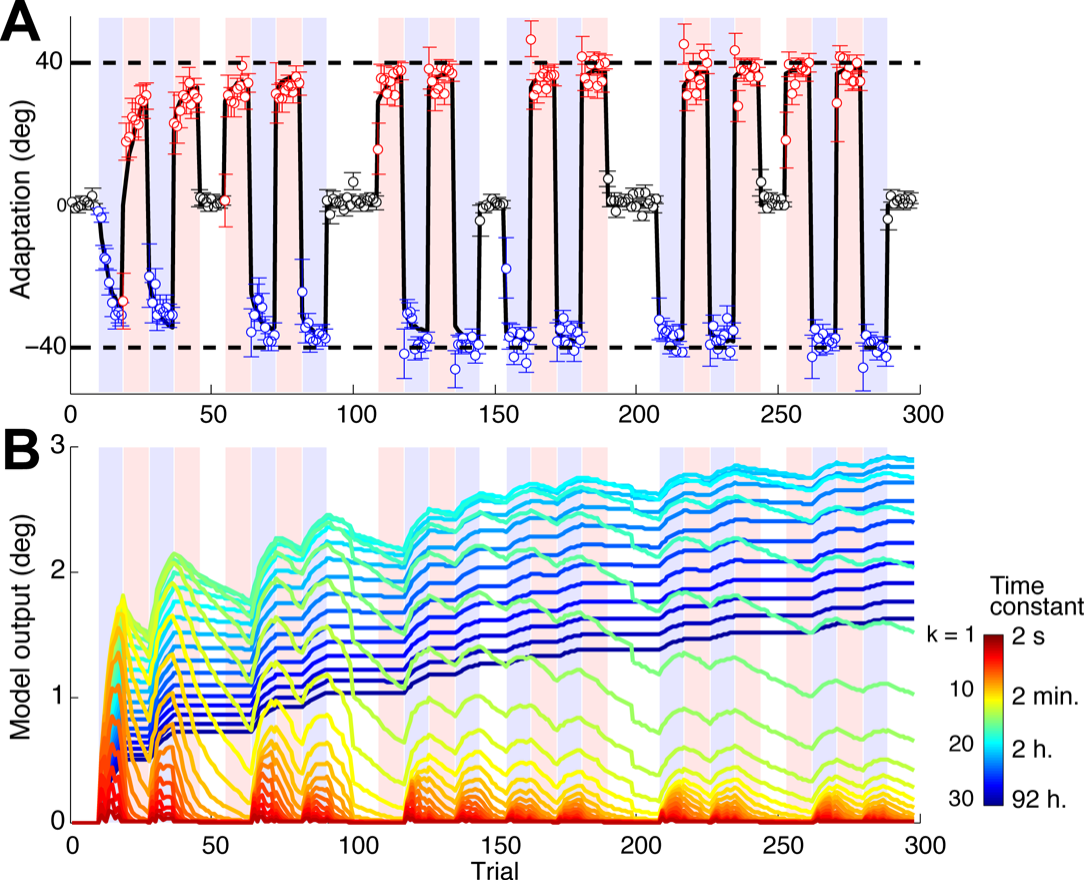
Mean subject adaptation and model fit. (A) Blue, red, and black circles indicate directions of joystick movements in Task 1 (40° rotation), Task 2 (−40°), and Control (0°), respectively, averaged across subjects (*N* = 21). Blue or red shaded regions indicate trials in Task 1 or 2, respectively. Regions with no shading indicate control trials. The thick black line indicates motor output, *y* of the multistate model (Eq 1). Error bars denote the standard error of the mean. (B) Trajectories of individual states of memory for Task 1, *x*_*k*,1_ (Eqs 1 and 2) of the fitted model. Colors indicate state numbers (*k* = 1,…,30) and corresponding time constants (*τ*_*k*_) as indicated by the color bar (see also S1 Table). Data of the model fit are available in S1 Data.

The overall mean adaptation level of 21 subjects showed that fast adaptation occurred within task blocks, and slow adaptation occurred across the task blocks for each task (Fig 2A). Forgetting across blocks, which can be observed by comparing the last trial of a task block and the first trial of the next block of the same task, gradually decreased across blocks. Thus, visual inspection of the behavioral data suggests the existence of multiple timescales in motor memory, initially dominated by faster memories and eventually dominated by slower memories.

We modeled adaptation as the sum of multiple memory states with different time constants. As in a previous model [5], a continuous distribution of timescales was approximated by multiple time constants. We defined 30 time constants, ranging from 2 s to ~92.6 h, in a logarithmic scale (S1 Table). In addition, the contextual cue (here the target color) selects the states for a specific task, as in a previous study [2]. Thus, the motor output at each trial *n* is given by

$$y(n)=\sum_{k=1}^{30}x_{k}{\left(n\right)}^{T}c(n),$$ (1)

where **x**_*k*_ = [*x*_*k*,1_ *x*_*k*,2_] with time constant *τ*_*k*_ (*k* = 1,…,30). The contextual cue vector is **c** = [1 0]^*T*^ for Task 1 and **c** = [0 1]^*T*^ for Task 2. This model thus assumes no interference between two tasks, i.e., perfect switching (please refer to S1 Text for the rationale of this choice of a model with no interference).

The states were updated by the error feedback, *e* = *f* − *y*, where *f* is, at each trial, one of the two visuomotor rotations, 40° or −40°. Because the intertrial interval (ITI) was random (see details in Materials and Methods), we modeled time decay as an exponential function of time [27]. The update equation from trial *n* to *n*+1 for the state of motor memory with time constant *τ*_*k*_ is thus given by

$$x_{k}(n+1)=x_{k}(n)e^{-T(n)/\tau_{k}}+\beta_{k}\cdot e(n)\cdot c(n),$$ (2)

where *T*(*n*) is the ITI following trial *n* and *β*_*k*_ is the learning rate. The learning rates depend inversely on the time constant *τ*_*k*_, as follows:

$$\beta_{k}=\frac{r}{\tau_{k}{}^{q}},$$ (3)

where *r* and *q* are strictly positive free parameters. As a result of this relationship, states with smaller time constants decay faster but are more rapidly updated [5,28].

Using averaged data of 21 subjects in actual adaptation to Tasks 1 and 2 (see details in Materials and Methods), the best-fitted parameters of the proposed model were *r* = 0.0333 and *q* = 0.201. The model fit is shown in Fig 2A, and the 30 states of Task 1 used in the fit are shown in Fig 2B. The overall fit to the averaged data was excellent (root mean squared error = 4.96°, R^2^ = 0.981). In addition, the fit to individual subjects’ data was satisfactory overall, although the fit was only modest for some subjects (mean ± SEM across subjects: R^2^ = 0.832 ± 0.189). We thus used the regressors calculated from the averaged adaptation data for the subsequent fMRI analysis.

### Model-Based Regression of fMRI

Because the multiple memory states are highly correlated with each other, especially for larger time constants (for instance, the correlation coefficient between the state with *τ*_21_ = 2.2 h and the state with *τ*_30_ = 92.6 h was 0.994), we first entered the states as single regressors in independent univariate analyses of blood-oxygen-level dependent (BOLD) signal (see Materials and Methods).

**Fig 3:**
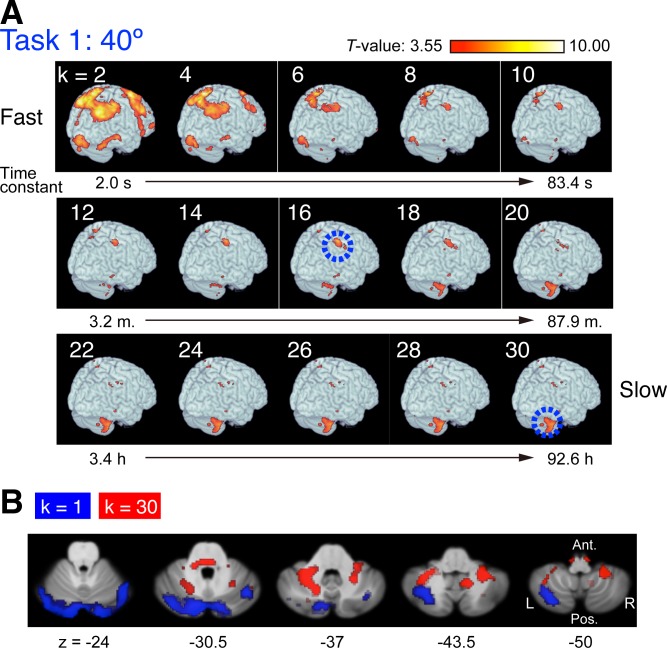
Correlated regions for individual states of motor memory with different time constants. (A) Red-yellow regions indicate regions where BOLD signal time courses were significantly correlated with individual states of motor memory (Fig 2B) (*p <* 0.001 uncorrected for multiple comparisons, see Materials and Methods). Color-coded *T*-values of regression coefficients are rendered on the right posterior view of the brain surfaces. The two blue circles indicate the anterior regions of the inferior parietal lobe and the cerebellum, which are consistently responsible for intermediate (*k* = 11,…,20) and slow states (*k* = 21,…,30). s: second, m: minute, h: hour (see also S1 and S2 Videos). (B) Regional difference in the cerebellum between the fastest (*k* = 1) and slowest (*k* = 30) states. Regions related to each state are indicated in the transverse sections from the superior (the left panel) to the inferior (the right panel) sections (see also S1 Fig). Data of the statistical maps are available in S2 Data.

Overall, this univariate model-based regression analysis revealed distinct patterns of regions for states with increasing time constants (Fig 3A). The faster states (*τ*_*k*_, ranging from 2.0 to 4.6 s; *k* = 1, 2, and 3) correlated mainly with activity in large regions in the frontal and parietal cortices and with activity in regions in the posterior-lateral cerebellum (see below). By contrast, the states with intermediate time constants (*τ*_*k*_, ranging from 2.1 to 87.9 min; *k* = 11,…,20) correlated with activity in a restricted area in the right anterior region of the inferior parietal lobe (aIPL, indicated by a blue circle in Fig 3A), which is the most anterior of the intraparietal sulcus. Note that the aIPL activity was prominently found in the right hemisphere, contralateral to the left hand used to perform the task but that weak activity was found in the left hemisphere when the threshold was lowered (see Discussion). The slower states (*τ*_*k*_, ranging from 2.2 to 92.6 h: *k* = 21,…,30) primarily correlated with activity in the anterior-medial cerebellum (see below). Supplementary videos show the patterns of correlated regions in both hemispheres for all time constants and corresponding regressors (S1 and S2 Videos). These patterns for each time constant were similar in both the 40° and the −40° condition.

Table 1 summarizes prominent clusters for the small (*k* = 1), middle (*k* = 16), and large (*k* = 30) time constants for Task 1. Significant clusters were found to be related to the fastest state (*k* = 1) in the parietal, frontal, and cerebellar regions when thresholded at *p* < 0.05 corrected for multiple comparisons (family-wise error rate [FWER]) throughout the brain at cluster level. Activity related to the middle state (*k* = 16) was significant in the aIPL (*p <* 0.05) when we applied small volume correction for the superior and inferior lobules, in line with previous studies reporting parietal contribution to visuomotor adaptation [9,15]. Activity related to the slowest state (*k* = 30) was significant in the left and right cerebellum at *p <* 0.05 corrected for the entire brain at cluster level. Fig 3B shows regional difference in the cerebellum between the fast (*k* = 1) and the slow (*k* = 30) states. Regions related to the fast state (blue) distribute in the posterior-lateral parts of the cerebellar cortex. These parts are mainly in the left and right crus 1 (see Table 1), which is connected with the prefrontal-parietal networks [29]. Note that activity was found in crus 1 not only for the fastest (*k* = 1) but also for relatively fast (*k* = 2,…,7) states (S1 Fig). By contrast, regions related to the slow state (red) distribute in the anterior-medial parts (mainly lobule 6 and partly lobule 8), which are connected with cerebral sensorimotor networks [30].

**Table 1:** Clusters identified by model-based regression analysis.

	Peak (MNI)			Cluster Size	*T*-Value at Peak	Corrected *p*-Value
	*x*	*y*	*z*			
**Fast state (*k* = 1)**						
Parietal clusters						
L precuneus	-4	-48	76			
R angular gyrus	42	-68	46	2616^a^	9.24	< 0.001
R precuneus	4	-56	74			
L angular gyrus	-40	-66	50	650	7.69	< 0.001
Prefrontal cluster						
R superior frontal gyrus	6	26	58	1474^b^	7.46	< 0.001
R middle frontal gyrus	50	24	40			
Cerebellar clusters						
L cerebellum crus 1	-12	-82	-28	413	6.69	< 0.001
L cerebellum 7b	-26	-74	-48	102	6.04	0.007
R cerebellum crus 1	18	-90	-22	84	5.96	0.014
Temporal clusters						
L inferior temporal gyrus	-50	-56	-8	79	6.02	0.017
R inferior temporal gyrus	54	-66	-12	225	6.74	< 0.001
R middle temporal gyrus	62	-42	-2	88	5.49	0.012
Precentral cluster						
L precentral gyrus	-44	4	42	92	5.66	0.010
**Middle state (*k* = 16)**						
R inferior parietal lobule	46	-38	56	25	5.30	0.013^c^
**Slow state (*k* = 30)**						
L cerebellum 6	-24	-44	-36	122	6.19	0.003
R cerebellum 8	26	-42	-52	75	5.89	0.017

^a^This cluster spans L precuneus, R angular gyrus and R precuneus.

^b^This cluster spans R superior frontal gyrus and R middle frontal gyrus.

^c^corrected for parietal (the superior and inferior parietal) lobules, otherwise corrected for the entire brain

Results from the univariate regression analysis with the 30-state model provided highly redundant but rich information on possible brain regions related to formation of multiple motor memories. For comparison, we checked if the standard two-state model [1,28] could explain our behavioral data and brain activity (see Materials and Methods). We found that the two-state model was a subset of the 30-state model. That is, the time constants of the fast process and the slow process in the two-state model were respectively 47.9 s and 1.5 h. These are very close to two time constants of the 30-state model, *k* = 9 (55.2 s) and *k* = 21 (2.22 h), with correlation coefficients of the states between the two models being respectively 0.993 and 0.991. Brain activity identified by the two-state model was essentially the same as that found by the states of *k* = 9 and *k* = 21 in the 30-state model (S2 Fig).

A possible concern with these univariate regression analyses is that the high-pass filtering with 128 s cut-off frequency, which is used in preprocessing to remove low-frequency noise due to scanner drift, could filter out the lower-frequency components in the cerebellum. An analysis of the frequency components of high-pass filtered BOLD signals using Fast Fourier Transform (FFT) shows that the frequency components in the cerebellum remained large enough to be correlated with the regressors with the slower time constants (see S3 Fig). A second possible concern with this analysis is that the identified activity is not related to memory states, but to errors. This is an especially valid concern for the faster states, because the memory states of the fast components correlate with the errors used for updating adaptation, with high values at the initial stages and low values at the late stages of adaptation. In our regression analysis of memory states, we included parametric regressors associated with error as those of no interest, i.e., as nuisance regressors for the decreasing effects of error-related activity on the estimation of memory-related activity. We verified that these error-associated nuisance regressors appropriately explained away the error in the regression analyses for the faster states (see S2 Text and S4 Fig).

### Dimensionality Reduction

**Fig 4:**
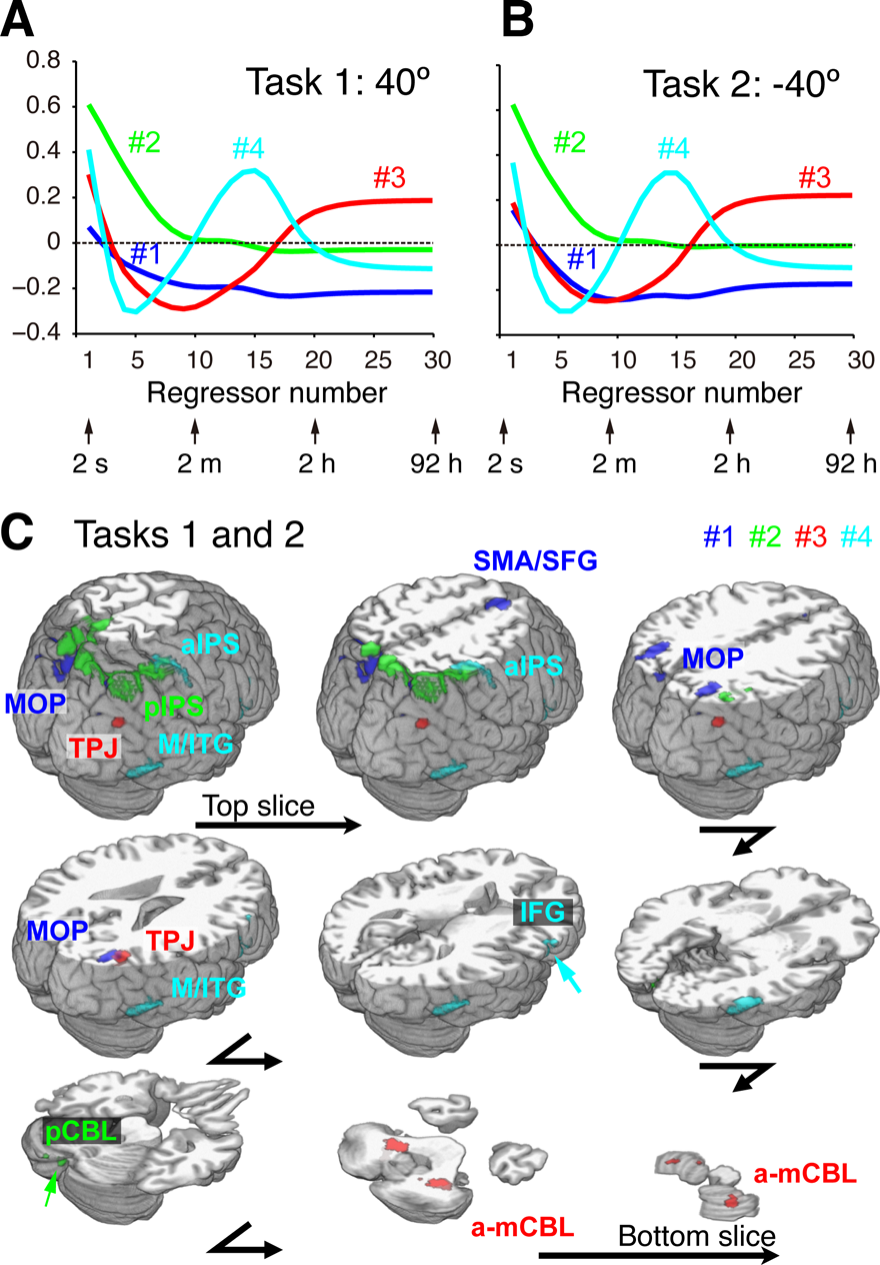
Eigenvariates and eigenimages of brain activity related to different timescales of sensorimotor memory. (A) and (B) Eigenvariates as a function of regressor number corresponding to different time constants for Tasks 1 and 2, respectively. (C) Eigenimages rendered on the brain surface and transverse slices at different levels. Eigenimages are thresholded so that the top 10% of the voxels are included for each component. Overlapped images between Tasks 1 and 2 are rendered (see S6 Fig and S2–S9 Tables for each task). SMA/SFG, supplementary motor area/superior frontal gyrus; MOP, medial occipitoparietal regions; aIPS, anterior part of intraparietal sulcus; pIPS, posterior part of intraparietal sulcus; TPJ, temporoparietal junction; IFG, inferior frontal gyrus; M/ITG, middle/inferior temporal gyri; a-mCBL, anterior-medial cerebellum; pCBL, posterior cerebellum. Data of *T*- values, to which the SVD analysis was applied, are available in S3 Data. Data of eigenvariates in Fig 4A and 4B are available in S4 Data. Data of eigenimages in Fig 4C are available in S5 Data.

The *T*-map shown in Fig 3 is redundant because of highly correlated regressors. We thus applied the sparse SVD to the *T*-value profiles of voxels as function of time constants to extract principal components (see Materials and Methods for details). All the voxels that survived the voxel-level threshold *p <* 0.001 for at least one time constant in the initial exploratory regression analysis were included in this sparse SVD analysis. The SVD analysis decomposed the data matrix into the following three matrices: eigenvariates, eigenvalues, and eigenimages. For both Tasks 1 and 2, a sparse SVD model with four components was selected via the model selection method of Bayesian Information Criterion (BIC) [31]. The contributions of these four components to the variance in the matrix of *T*-values were (54.26%, 38.94%, 6.32%, 0.47%) and (51.38%, 39.72%, 8.35%, 0.55%) for Tasks 1 and 2, respectively. The first and second eigenvariates correspond to the fast states (the second eigenvariate has notably large values for small time constants that rapidly approach zero around 2 min), while the third and fourth eigenvariates represent the slow and middle states, respectively (Fig 4A and 4B). Because a similar pattern was observed in corresponding eigenimages for Tasks 1 and 2 (see S5 Fig and S1 and S2 Videos), their overlap is presented in Fig 4C after each image was thresholded so that the top 10% of voxels with the highest values are included in the image. The first component is located mainly around the junction between the supplementary motor area and superior frontal gyrus (SMA/SFG), and in medial occipitoparietal regions (MOP). The second is located mainly in the posterior region of the intraparietal sulcus (pIPS) and partly in the posterior cerebellum (pCBL: crura 1 and 2). The third is mainly in the anterior-medial part of the cerebellum (a-mCBL: lobules 6 and 8), and the right temporoparietal junction (TPJ). The fourth is in the anterior part of the intraparietal sulcus (aIPS), which includes the aIPL region for the middle component (e.g., *k* = 16), the middle temporal and inferior temporal gyri (M/ITG), and the inferior frontal gyrus (IFG). More detailed quantification of eigenimages for Tasks 1 and 2 are provided in S2–S9 Tables.

We then verified whether a reduced model with four states derived from the SVD analysis, instead of the full model with 30 states, could account for the behavioral adaption. From the eigenvariates of the *T*-map (Fig 4A and 4B), we constructed a reduced four-state model consisting of "eigenstates," each of which was estimated as a linear combination of the 30 regressors weighted by the corresponding eigenvalues. The variance explained by the four-state model was as high as the original 30-state model (mean squared error = 4.96°, *R* ^2^ = 0.981). Thus, the model reduced from 30 to four states based on the neural activities well explains the behavioral data.

### Decoding Analysis

**Fig 5:**
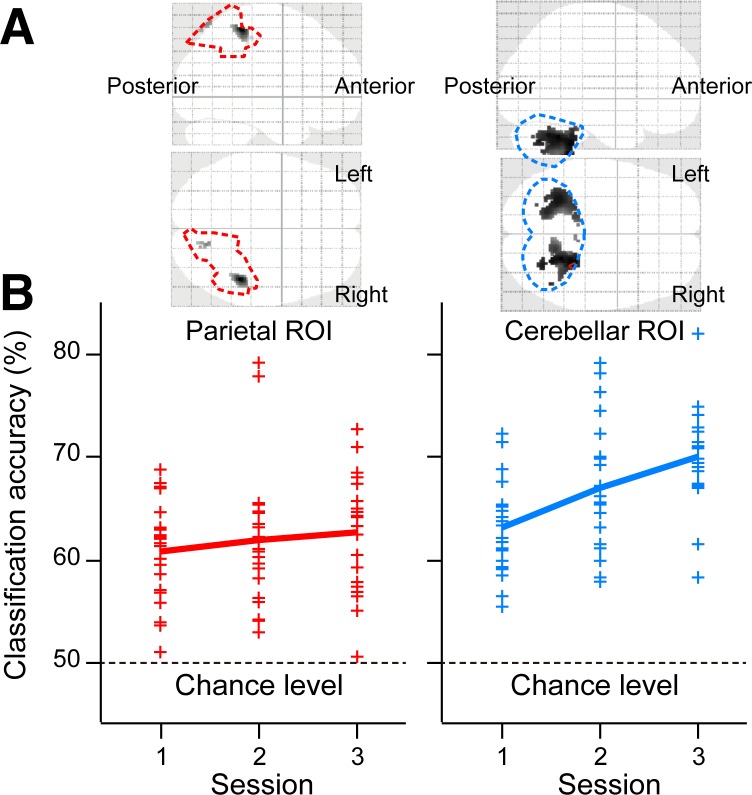
Regions of interest (ROIs) and classification accuracy of multivoxel pattern analysis. (A) Functional ROIs to which multivoxel pattern analysis was applied (gray-black regions). Broken lines indicate anatomical ROIs (red: the superior and the inferior parietal lobes; cyan: the cerebellum). Left and right panels indicate the parietal and cerebellar ROIs, respectively. Top and bottom panels show the regions projected to the sagittal and the transverse planes, respectively. (B) Classification accuracy of Tasks 1 and 2 as a function of sessions using activity patterns in the above functional ROIs. A plus (+) marker indicates accuracy averaged within each subject according to cross-validation tests (see Materials and Methods). Thick lines indicate accuracy averaged across subjects. Data of the individual classification accuracy are available in S6 Data.

The above model-based regression analysis indicated contributions of the parietal and the anterior cerebellar regions to the middle and slow states, respectively. To exclude the possibility that these regression results are due to spurious correlations, we then conducted a decoding analysis to test whether the regional brain activity could be used to classify the two rotations (40° and −40°). If classification accuracy varies across the three sessions, this would indicate that activity in these regions changes with dynamics similar to the dynamics of medium or slower states. We thus applied a multivoxel pattern analysis (MVPA) to parietal regions in which BOLD signals significantly correlated with at least one of the intermediate components (*k* = 11,…,20), and in cerebellar regions in which signals were significantly correlated with at least one of the slow components (*k* = 21,…,30) (Fig 5A, see Materials and Methods). The MVPA revealed significantly above chance accuracy (50%) in the right aIPL as well as in the cerebellum for all sessions, with averaged accuracy across subjects ranging from 60% to 70% (Fig 5B). This above-chance classification is not surprising because the direction of hand movements changed depending on the rotation types. However, two-way analysis of variance (ANOVA) with regions of interest (ROIs) and sessions as a within-subject factor revealed a significant interaction between the two factors (*F*_(2, 40)_ = 3.504, *p <* 0.05). A simple main effect analysis revealed significant increase of accuracy across sessions in the cerebellum (*F*_(2, 80)_ = 10.16, *p <* 0.001), but no significant difference in the right aIPL (*F*_(2, 80)_ = 0.736, *p* = 0.482). These results thus indicate that specificity of activity patterns to the task (40 or −40° rotations) increased with sessions in the cerebellum but did not change in the parietal regions. We then verified that the increase in classification accuracy across sessions observed in the cerebellum was unlikely due to behavioral confounds during adaptation (see S3 Text).

## Discussion

We investigated the spatiotemporal neural correlates of motor memory involved in visuomotor adaptation via estimation of the latent memory states derived from a model with multiple states with different time constants. A univariate regression analysis, which correlated the model states with brain activity during the whole adaptation process, first located the neural substrates related to formation of the multiple motor memories. Then, a sparse SVD analysis showed four characteristic networks, associated with a specific profile of correlation with different time constants. Finally, a classification analysis showed that specific activity patterns to the rotation type were acquired in the cerebellum as adaptation proceeded.

We organize the following discussion of our results from the faster to the slower time constants. For the first few fastest constants (2 s to 4.6 s), various brain regions were activated, including frontal and parietal lobes, as well as the visual cortex, the temporal cortex, and regions in the posterior part of the cerebellum, specifically in crus 1. It is known that regions in the crus 1 are connected with prefrontal and parietal cerebral regions according to studies on cerebro-cerebellar connections in monkeys [29] and humans [30]. For slower but still relatively fast time constants up to *k* = 6 (15.9 s), the widespread activated regions became more localized into the PPC. A possible reason for activation of parietal activity is mental rotation, which has been known to contribute to early adaptation [7,32,33] and has been consistently localized in the superior parietal regions (e.g., [34,35]). In line with these studies, the subjects in our study who showed larger reaction times tended to perform the task better with lower directional errors (*R* ^2^ = 0.229, *p* = 0.028).

For intermediate time constants around 16.7 min, we found a characteristic region of activation in the right aIPL (blue circle in Fig 3). This is consistent with a prism-adaptation study [6] finding activity contralateral to the reaching hand in the lateral bank of the intraparietal sulcus, close to the aIPL activity in our study (note that subjects used the left hand in the current experiment). It has been suggested that the left parietal regions are critical for visuomotor rotation, because patients with left parietal damage show a deficit in adaptation [36,37]. We also found activity in the left aIPL correlated with intermediate time constants if the threshold is lowered (*p <* 0.05 uncorrected), but the activity in the right aIPL was more significant than that in the left aIPL. Muhta and colleagues showed the importance of the left parietal region for construction of visuomotor mapping based on online correction of error [36]. In contrast, we only provided terminal feedback after the end of joystick movement, and the role of online correction was relatively small. Thus, although further studies are needed to understand the contradiction in parietal laterality between our study and Mutha et al.’s, the above difference in error feedback may explain this difference.

For slower time constants, the number of correlated voxels in the aIPL decreased, and the number in the anterior-medial cerebellum increased. With time constants longer than 1 h, the main activities were identified in the anterior-medial cerebellum; this result is in line with previous studies [6,15,18]. Regions related to the slow states distribute in the anterior-medial parts and mainly in lobule 6. These cerebellar regions are connected with cerebral sensorimotor networks, including the primary motor and sensory cortices, the premotor cortex, and the supplementary motor area [30]. This suggests that the cerebellar slow states directly contribute to sensorimotor control without help from cognitive processes (prefrontal-parietal functions) probably corresponding to an “autonomous” stage [38] by constructing internal models [18,25,39]. A recent study reported that transcranial direct current stimulation (tDCS) over the cerebellum induced faster adaptation during training but did not affect retention after training [40]. Because tDCS is likely to affect neural activity in the posterior part of the cerebellum to a greater extent than in the anterior-medial part, our findings of the fast components of motor memory in the posterior part (Fig 3 and S1 Fig) are consistent with this previous study. The existence of slow components in lobule 6 is consistent with a study reporting that patients with focal degeneration in this lobule have difficulty in adapting to a visuomotor rotation [41]. In addition, activity related to kinematic errors that drive visuomotor rotation has been found in cerebellar regions, including lobule 6 [20].

The SVD analysis of variability in specificities to timescales over the brain identified four components, two fast, one middle, and one slow, each associated with different brain networks. Three groups of components (fast, middle, and slow) are consistent with a recently proposed three-component model of visuomotor adaptation [42]. The first and second SVD components are subset regions of the fastest component delineated by the regression analysis and indicate the existence of two types of fast components: one related to SMA/SFG and MOP, and the other related to posterior IPS and cerebellum. Our previous study [43] indicated that medial parietal regions (including MOP) are related to switching of internal models based on contextual cues and that posterior-lateral regions of the IPS are related to switching based on sensorimotor feedback. Analogy of the current results to our previous study suggests that the first SVD component corresponds to association between visual cue (target color) and responses for tasks, while the second one corresponds to fast adaptation (or rearrangement of haptic directions) based on sensorimotor feedback. The third component confirmed the slow component in the anterior-medial cerebellum. The fourth component in the aIPS completely includes aIPL found for the middle time constant. This component was also found in the IFG and M/ITG. Neurons in the IFG are activated when monkeys [44]and humans [45,46] observe goal-directed hand actions and when humans imagine hand actions [47]. The IFG has been suggested to contain sensorimotor memory representation related to hand movement [48]. The M/ITG is known to be involved in visual motion analysis [49], but specific interpretation of this region in the visuomotor adaptation is unknown, at least to our knowledge.

Our decoding analysis indicated that the activity pattern in the cerebellum became more specific to rotation type as adaptation proceeded. A previous study by one of us [25] showed that cerebellar activities correlated with learning to control two different cursors (rotation and velocity) were spatially segregated, supporting modular organization of internal models, thus suggesting that overlapped regions represent common properties of learning two tasks. In contrast, the results of the present study show no significant regional difference of activities in either the parietal or the cerebellar regions between visuomotor rotations of 40° and −40°(S6 Fig). We surmise that the overlap arises because the two visuomotor transformations are identical except for the rotation angle. Within this common cerebellar region, as well as in the PPC, the MVPA discriminated the representation of the opposing rotations with higher decoding accuracy than chance level. As mentioned earlier, this result is not surprising because the direction of hand movements changed depending on the rotational types and could contribute to successful decoding. Importantly, however, we observed significant increase of the decoding accuracy across sessions in the cerebellar regions related to the slow states (lobules 6 and 8), but not in the parietal regions related to middle states. The increase in classification accuracy in the cerebellum, especially from the second to the third session, does not appear to be due to changes in performance, because we could not identify significant difference in performance between the second and third sessions.

While the primary motor cortex (M1) has been involved in the late stage of adaptation in previous studies [8,40,50,51], we found no significantly correlated activities in M1. In addition, in a recent study [51], we found that MVPA could classify opposite rotational types (90° or −90°) from activity patterns in sensorimotor cortex including M1, suggesting separate representation for dual visuomotor adaptation. However, the classification was based on fMRI activity measured after intensive training on a continuous tracking task for more than 3 d and a total of 160 min. Therefore, M1 may be correlated with even slower timescales beyond the range of our study, inducing structural changes [8], although further studies would be necessary to confirm this correlation.

Unlike conventional fMRI regression analysis, model-based fMRI regression analysis allows the study of the underlying latent variables generating the behavior in motor adaptation. In previous neuroimaging studies of motor adaptation, the observable behavioral variables of interest were used to define contrasts or regressors for analysis of brain activity. However, the multiple motor memories and related activity that drive the behavior are internal to the subject undergoing adaptation and thus cannot be measured directly (although they can, in theory, be manipulated by experimental conditions such as task schedules, e.g., [52]). Here, as in a number of reinforcement learning studies (e.g., [53] for review), we circumvented this difficulty by first estimating internal memory states via computational modeling and then by using these internal variables in the regression analysis to detect neural representations related to formation of motor memories at multiple timescales. Note that we carefully designed our regression models by including possible confounding variables, notably type of task, hand movement, error, and reaction time in each trial. However, when confounding variables are correlated with memory states, such as errors with fast memory states, regression cannot completely dissociate activity related to these confounding variables from activity directly related to memory itself. In addition, our regression models did not include additional behavioral and physical quantities that may correlate with memory states in the model and that may or may not be related to formation of multiple memories, such as attention, eye movements, and repetition of the task. Thus, our results revealed the neural substrates related to formation of the multiple memories at multiple time scales, but not necessarily the neural substrates of multiple memories at multiple time scales per se. Experiments in which nuisance parameters (such as error) are varied while adaptation is constant would allow the effects of these confounds to be dissociated.

Recent studies have suggested that motor adaptation is a multifaceted process. In particular, behavior during adaptation is not only updated by error-based learning mechanisms, as we have assumed with our model, but also presumably updated by reward-based and use-dependent mechanisms [54–56]. Each of these processes likely operates at multiple time constants as well. In addition, explicit and implicit aspects of motor adaptation have been recently shown to have fast and slow dynamics, respectively [57]. Thus, although the interpretation of what “memory states” represent varies between adaptation studies, our experiment of the neural correlates related to formation of motor memories at multiple time scales is, we believe, highly relevant. Note, however, that our study does not provide a clear picture of the connectivity and spatial arrangements of the multiple neural representations involved. In particular, in line with a previous study supporting a parallel architecture of motor memories over a serial architecture [2], we have assumed a parallel architecture in our model in which all memories were updated by a common error signal (see Eq 2). Our results, however, cannot provide evidence for such parallel architecture. To further clarify the actual neural mechanism, model-based fMRI regression can be complemented by functional connectivity analysis [58] or causal and interfering manipulation of neural function via transcranial magnetic stimulation (TMS) or tDCS [59–62].

## Materials and Methods

### Subjects

Twenty-one right-handed and neurologically healthy volunteers participated in the study (20–50 y old, mean age of 27.3 y, six females). Handedness was assessed by a modified version of the Edinburgh Handedness Inventory [63]. Written informed consent was obtained from all subjects in accordance with the Declaration of Helsinki. The experimental protocol received approval from the local ethics committee at the Advanced Telecommunications Research Institute International.

### Task Procedures

We designed a dual-task adaptation experiment with two opposing visuomotor rotations. At the beginning of each trial, a white cross appeared at the center of screen; this cross served both as the fixation point and as the initial cursor position. A round colored target of 0.7 cm radius appeared on the top of the screen 8 cm from the center. Subjects were instructed to manipulate an fMRI compatible joystick to move the cursor to the target within 1.5 s (maximum movement time); otherwise, the trial was considered a missed trial, and the data was not analyzed. After the maximum movement time, the cursor appeared in the direction of the joystick movement at 8 cm from the center for 500 ms to provide angular error feedback. To encourage subjects to respond faster, the color of the feedback cursor turned yellow if the subject did not move within 800 ms. ITIs were randomly generated from 4 to 14 s from an exponential distribution, in 2 s increments. For each trial, we calculated the angular error between the target direction and the final cursor direction from the center of the screen. Note that the size of the target was equivalent to 10° in visual angle, allowing up to ±5° of error to “hit” the target.

There were three different tasks: a control task and the two different visuomotor tasks, Tasks 1, 2, in which the cursor movement was rotated 40° and −40°, respectively. In the control task, the cursor movement was not rotated. The experiment was divided in three sessions, each session lasting about 11 min, with a 1-min break between sessions. Each session consisted of 99 trials, with 27 trials for the control task and 36 trials for each of Tasks 1 and 2. Three different target colors, red, blue, and green, were used to distinguish the different tasks. Each task was presented in blocks of nine trials, with blocks presented according to schedules such as C1212C2121C2121C1212C, where C, 1, and 2 indicate a block of nine trials for the Control, Task 1, and Task 2. There were two possible schedules starting with either Task 1 (11 subjects) or 2 (10 subjects), because we counterbalanced the sequence of Tasks 1 and 2 across subjects and sessions to eliminate any confounding effects due to schedule. Similarly, target colors were counterbalanced across subjects. Before the experiment, the participants performed a familiarization session of 150 trials of the control task.

Stimuli were presented on a liquid crystal display and projected onto a custom-made viewing screen. Subjects laid in a supine position in the scanner, viewed the screen via a mirror, and were unable to see their hand throughout this task. They were instructed to use their left thumb and index/middle pair fingers to control the joystick with the left upper arm immobilized using foam pads to minimize body motions.

### Model Fitting

We used the MATLAB *fmincon* function to estimate the value of the two parameters *r* and *q* that minimize the mean squared error between the actual adaptations of subjects for Tasks 1 and 2 and model predictions, *y*(*n*) (see Eqs 1 and 3). The adaptation data used for the model fit were calculated by averaging the observed adaptations of 21 subjects, excluding missed trials and trials with large (>40°) overshoot. Less than 1% of the total number of trials was excluded. Because of the task sequence counterbalancing, the average was computed after inverting the sign of adaptation for ten subjects starting with Task 2.

Using the estimated parameters, we simulated the time series of the 30 states of memory for each task, **x**_*k*_ every 1.8 s, corresponding to scanner repetition time (TR). Because the model equation (Eq 2) updates the states at each trial, we interpolated and resampled the states of memory at the time of image acquisitions, i.e., multiples of TR by calculating the decay depending on time constants following trial *n*. The 30 simulated memory traces for each task were used as regressors for the univariate fMRI analysis. For additional modeling with the standard two-state model [1,28], we fitted four free parameters, time constants and learning rates for the fast and the slow processes, using the same method described above. It is notable that the 30 time constants in the proposed model were predetermined with a logarithmic scale and the 30 learning rates were calculated by two free parameters (Eq 3). The estimated states of the fast and the slow processes were compared with those of the proposed model (see Results and S2 Fig).

### MRI Acquisition

A 3-T Siemens Trio scanner (Erlangen, Germany) with a 12-channel head coil was used to perform T2*-weighted echo planar imaging (EPI). A total of 368 scans were acquired for each session with a gradient echo EPI sequence, and each subject underwent three sessions. The first five scans were discarded to allow for T1 equilibration. Scanning parameters were repetition time (TR), 1,800 ms; echo time (TE), 30 ms; flip angle (FA), 70°; field of view (FOV), 192 × 192 mm; matrix, 64 × 64; 30 axial slices; and slice thickness, 5 mm without gap. T1-weighted anatomical imaging with an MP-RAGE sequence was performed with the following parameters: TR, 2,250 ms; TE, 3.06 ms; FA, 9°; FOV, 256 × 256 mm; matrix, 256 × 256; 192 axial slices; and slice thickness, 1 mm without gap.

### Processing of fMRI Data

Image preprocessing was performed using SPM8 software (Wellcome Trust Centre for Neuroimaging, http://www.fil.ion.ucl.ac.uk/spm). All functional images were first realigned to adjust for motion-related artifacts. The realigned images were then spatially normalized with the Montreal Neurological Institute (MNI) template and resampled into 2-mm-cube voxels with sinc interpolation. All images were spatially smoothed using a Gaussian kernel of 8 × 8 × 8 mm full width at half maximum. The smoothing was not performed for multivoxel pattern analysis (see below), as this could blur fine-grained information contained in multivoxel activity [64].

### Model-Based Regression Analysis of fMRI Data

We first conducted a model-based regression analysis of fMRI data. For each of Task 1 and Task 2, the 30 memory traces with different time constants, which were estimated with the previous behavioral modeling, were used as explanatory variables (i.e., regressors) using the general linear model (GLM). To accommodate the problem of multicollinearity due to similarity of regressors between adjacent time constants, we separately estimated 30 regression models corresponding to individual time constants:

$$S=\alpha_{1}x_{k,1}+\alpha_{2}x_{k,2}+(Effectsofnointerests)+\varepsilon ,$$ (4)

Here, *S* is a time series of the BOLD signal at each voxel. The regressors (*x*_*k*,1_ and *x*_*k*,2_) in each model are time series of motor memories corresponding to one of the 30 time constants (*k* = 1,…,30; see Eq 1 in behavioral results and modeling) for Tasks 1 (40°) and 2 (−40°). Each of them was resampled at scan timings of brain activity and orthogonalized using an SPM function (spm_orth.m).

We included the following regressors as “effects of no interests” in the analysis. First, pulse functions that were assigned 1 at every onset of joystick movement and 0 otherwise were included to model hand movements. We assumed that convolution of these functions with a canonical hemodynamic function can model the hand movements with a short movement time in our task (mean ± SEM: 296 ± 2.50 ms), with a separate model for each trial type (Tasks 1 and 2 and the Control). In addition, two parametric regressors were included to model the effect of directional errors and reaction times (“parametric modulation” in SPM). In each trial, these parametric regressors also used the pulse functions at onset of joystick movement, but their amplitudes were modulated by directional error and reaction time. We included three boxcar functions, each of which modeled a session effect. Therefore, 12 regressors in total (3 [hand movement, error and reaction time] x 3 [tasks] + 3 [sessions]) were included as effects of no interests.

Low-frequency noise was removed using a high-pass filter with a cut-off period of 128 s, and serial correlations among scans were estimated with an autoregressive model implemented in SPM8. Contrast images of each subject, generated using a fixed-effects model, were taken into the group analysis using a random-effects model of a one-sample *t*-test. Because the secondary purpose of the model-based regression analysis was to recruit possible regions related to many (30) states of motor memory for the sparse singular value decomposition analysis (see below), activation was reported with a lenient threshold of *p <* 0.001 uncorrected for multiple comparisons at the voxel level. In further analyses, we applied a stricter inclusion criterion, a cluster-level correction based on the FWER, to representative activations such as those related to the fast, middle, or slow states.

### Sparse SVD Analysis

The univariate analysis with a *p*-value cutoff resulted in a grand total of 23,413 and 24,676 selected voxels associated with 30 memory states for Tasks 1 and 2, respectively. Thus, the univariate analysis provided two matrices X of *T*-values for these selected voxels, each for one of the two tasks. We applied the sparse SVD [65] to each matrix X. The sparse SVD was implemented in a refined way with the orthogonality constraints. Specifically, we adopted the regularized estimator $\left(\hat{D},\hat{U},\hat{V}\right)$ that minimizes the sum of squared Frobenius norm of the difference between X and *UDV* ^*T*^ and a sparsity-inducing regularization term on matrices *D*, *UD*, and *VD*, subject to the orthogonality constraints that both matrices *U* (eigenvariates) and *V* (eigenimages) are orthonormal, where *D* (eigenvalues) is a diagonal matrix. We employed the entry-wise *L*_1_ norm, which is the sum of all absolute entries of a matrix, multiplied by a regularization parameter to regularize the three matrices *D*, *UD*, and *VD*. Each regularization parameter was chosen in a decreasing grid of 20 values ranging from 200 to 0.1 (equally spaced in the logarithmic scale). For each set of regularization parameters, we obtained a sparse SVD model $\left(\hat{D},\hat{U},\hat{V}\right)$ in which the number of nonzero singular values in $\hat{D}$ gives the rank of the matrix decomposition and singular vectors in $\hat{U},\hat{V}$ can be sparse with some entries being zero. This produced a sequence of SVD models with sparse singular values and vectors. We then employed the standard BIC model selection criterion [31] to select the sparse SVD model.

### Multivoxel Pattern Analysis (MVPA)

We additionally conducted an MVPA to test if the regional brain activity could be used to classify the two rotational types (40° and −40°). The ROIs include the right parietal lobe and the cerebellum. Our previous model-based regression analysis suggested that these regions are related to the middle (the parietal regions) and the slow (the cerebellum) states (blue circles in Fig 3A). The ROI of the right parietal region was the superior and the inferior parietal lobes according to anatomical map in Pick Atlas (http://fmri.wfubmc.edu/software/PickAtlas). The cerebellar ROI was anatomically defined bilaterally. Fig 5A shows the parietal and cerebellar regions enclosed by red and cyan curves, respectively. Within the parietal ROI, we applied MVPA to BOLD signals of voxels that were significantly correlated with at least one of the intermediate states (*τ*_*k*_, ranging from 2.1 to 87.9 min: *k* = 11,…,20) in the model-based regression analysis. In the cerebellum, MVPA was applied to signals that were significantly correlated with at least one of the slow states (*τ*_*k*_, ranging from 2.2 to 92.6 h: *k* = 21,…,30). The voxels selected in Tasks 1 and 2 were jointly used by taking a union.

To conduct the classification, we first modeled all 297 trials as separate pulse regressors at the onset of movement, which were convolved with a canonical hemodynamic response function. This analysis yielded 297 independently estimated parameters (beta values) for each individual voxel. The 198 trials with rotational conditions (40° or −40°) were subsequently used as inputs for the MVPA. The classification was performed with a linear support vector machine (SVM) implemented in LIBSVM (http://www.csie.ntu.edu.tw/~cjlin/libsvm/), with default parameters (a fixed regularization parameter C = 1). The separate training and testing datasets were generated with a pseudo-random half split of all the samples. Cross validation was then conducted for 1,000 times for each subject, and the average classification accuracy was estimated. The two-way ANOVA with ROIs and sessions as an intrasubject factor was used to test the differences in classification accuracies.

## Supporting Information

S1 Data The dataset contains adaptation data (mean with error bars) with model fits and individual memory states for Fig 2. To open this file, Matlab (Mathworks) is needed.
(ZIP)

S2 Data The dataset contains the SPM8 images of the group statistical *T*-value maps (Analyze format) for Fig 3. (ZIP)

S3 Data The dataset contains the *T*-values from which we extracted eigenvariates and eigenimages by using the SVD analysis for Fig 4. To open this file, Matlab (Mathworks, Inc.) is needed.
(ZIP)

S4 Data The dataset contains the eigenvariates for Fig 4A and 4B. (XLSX)

S5 Data The dataset contains the eigenimages (NIfTI format) for Tasks 1 and 2 for Fig 4C. (ZIP)

S6 Data The dataset contains the individual classification accuracy for Fig 5B. (XLSX)

S1 Fig Regions in the cerebellum that correlates with memory states with relatively fast time constants (*k* = 2,…,7). Highest correlations were found in the posterior region of the cerebellum for faster time constants.
(TIF)

S2 Fig Correlated regions for states of memory derived from two-state or 30-state model. (A) We fitted the behavioral data with a two-state model and obtained the fast and slow process with time constants of 47.9 s and 1.5 h, respectively. We conducted a regression analysis of brain activity using the fast and the slow processes. The regression model included the four regressors that corresponded to fast and slow components for Tasks 1 and 2 and other regressors modeling effects of no interest such as hand movements, error, and reaction time. Results were thresholded at a lenient statistical level (*p* < 0.01 uncorrected) for each task, and regions that overlapped between the two tasks are indicated by colors (blue for the fast and red for the slow component). (B) The time constant of the fast (47.9 s; blue curve in the left panel) and the slow (1.5 h; blue curve in the right panel) states are very close to those from the suggested 30-state model, *k* = 9 (55.2 s; red curve in the left panel) and *k* = 21 (2.22 h; red curve in the right panel)—see S1 Table; the correlation coefficients between corresponding states in the two models are respectively 0.993 and 0.991. (C) For comparison, we thresholded results for the two time-constants (*k* = 9 and *k* = 21) from the 30-state model at *p* < 0.01 (uncorrected) for each task and indicated the regions overlapped between Tasks 1 and 2. Results are similar to those of the two-state model.
(TIF)

S3 Fig Analysis of the frequency components of high-pass filtered BOLD signals using FFT. Here, we illustrate how the low-frequency components in the cerebellum were attenuated by the high-pass filter but remained sufficiently large to be correlated with the regressors with the slower time constants. Left panels show a representative BOLD signal in the right cerebellum (MNI: [22 −46 40]) for one subject (not averaged). Right panels show the mean and the SEM of frequency components (the line and the shaded region, respectively) across subjects for the same voxel. The top panels show the BOLD signal and its frequency components before and after high-pass filtering (using spm_filter.m with cutoff frequency: 0.0078 Hz, period: 128 s). As expected, the frequency components below the cut-off frequency (black dotted line) were attenuated; however, they were not completely eliminated. The middle panels show the residual signal from the BOLD signal and its frequency components not explained by a design matrix for the slow regressor (*k* = 30). The bottom panels show the signal explained by the design matrix and its frequency components. As can be seen on the bottom-right panel, the magnitude of the signal at low frequencies is still sufficiently large, resulting in significant correlation of the BOLD signals in the cerebellum with the slow regressor.
(TIF)

S4 Fig Brain activity associated with performance-error regressors (group analysis). (A) Activity derived from the regression analysis of the slowest component. (B) Activity derived from the analysis of the fastest component. Activity was thresholded at *p* < 0.001 uncorrected for multiple comparisons. The left figures represent activity projected to the sagittal, coronal, and transverse planes (glass brain). The right figures show activity projected to the surface of the brain from the left, posterior, and top viewpoints.
(TIF)

S5 Fig Eigenimages with multiple transverse sections for Tasks 1 and 2. As described in the main text, the correlated brain activities were characterized with the top four components, two relatively fast components (first and second), an intermediate component (third), and one slow component (fourth). A similar pattern was found for Tasks 1 and 2.
(TIF)

S6 Fig Effects of different tasks on brain activities. The correlated brain activities showed no significant difference between Tasks 1 and 2 in either the parietal (*k* = 16, *τ*_*k*_ = 16.7 min) or the cerebellar (*k* = 30, *τ*_*k*_ = 92.6 min) regions.
(TIF)

S1 Table The 30 time constants *τ*_*k*_ (*k* = 1,…,30) of the model following a logarithmic scale. (DOCX)

S2 Table Clusters in eigenimage of the first component (Task 1). (DOCX)

S3 Table Clusters in eigenimage of the second component (Task 1). (DOCX)

S4 Table Clusters in eigenimage of the third component (Task 1). (DOCX)

S5 Table Clusters in eigenimage of the fourth component (Task 1). (DOCX)

S6 Table Clusters in eigenimage of the first component (Task 2). (DOCX)

S7 Table Clusters in eigenimage of the second component (Task 2). (DOCX)

S8 Table Clusters in eigenimage of the third component (Task 2). (DOCX)

S9 Table Clusters in eigenimage of the fourth component (Task 2). (DOCX)

S1 Text Rationale for the choice of an adaptation model without interference. (DOC)

S2 Text Activity correlated with error-associated regressors. (DOC)

S3 Text Increased classification accuracy in the cerebellum is unlikely to be caused by behavioral confounds. (DOC)

S1 Video Correlated regions for individual states of motor memory and corresponding regressors with varying time constants (Task 1). (MOV)

S2 Video Correlated regions for individual states of motor memory and corresponding regressors with varying time constants (Task 2). (MOV)

## Abbreviations

aIPL: anterior region of the inferior parietal lobe
a-mCBL: anterior-medial part of the cerebellum
ANOVA: analysis of variance
BIC: Bayesian Information Criterion
BOLD: blood-oxygen-level dependent
EPI: echo planar imaging
FA: flip angle
FFT: Fast Fourier Transform
fMRI: functional magnetic resonance imaging
FOV: field of view
FWER: family-wise error rate
GLM: general linear model
IFG: inferior frontal gyrus
IPS: intraparietal sulcus
ITI: intertrial interval
M1: primary motor cortex
M/ITG: the middle temporal and inferior temporal gyri
MNI: Montreal Neurological Institute
MOP: medial occipitoparietal
MRI: magnetic resonance imaging
PET: positron emission tomography
PFC: prefrontal cortex
pIPS: posterior region of the intraparietal sulcus
pCBL: posterior cerebellum
PPC: posterior parietal cortex
ROI: region of interest
SEM: standard error of the mean
SFG: superior frontal gyrus
SMA: supplementary motor area
SVD: singular value decomposition
SVM: support vector machine
tDCS: transcranial direct current stimulation
TE: echo time
TPJ: temporoparietal junction
TMS: transcranial magnetic stimulation
TR: repetition time
