# Supplementary figures and images for: Neural Substrates Related to Motor Memory with Multiple Timescales in Sensorimotor Adaptation
Source: PLoS Biol. 2015 Dec 8;13(12):e1002312. doi: 10.1371/journal.pbio.1002312 (PMC4672877; doi:10.1371/journal.pbio.1002312)

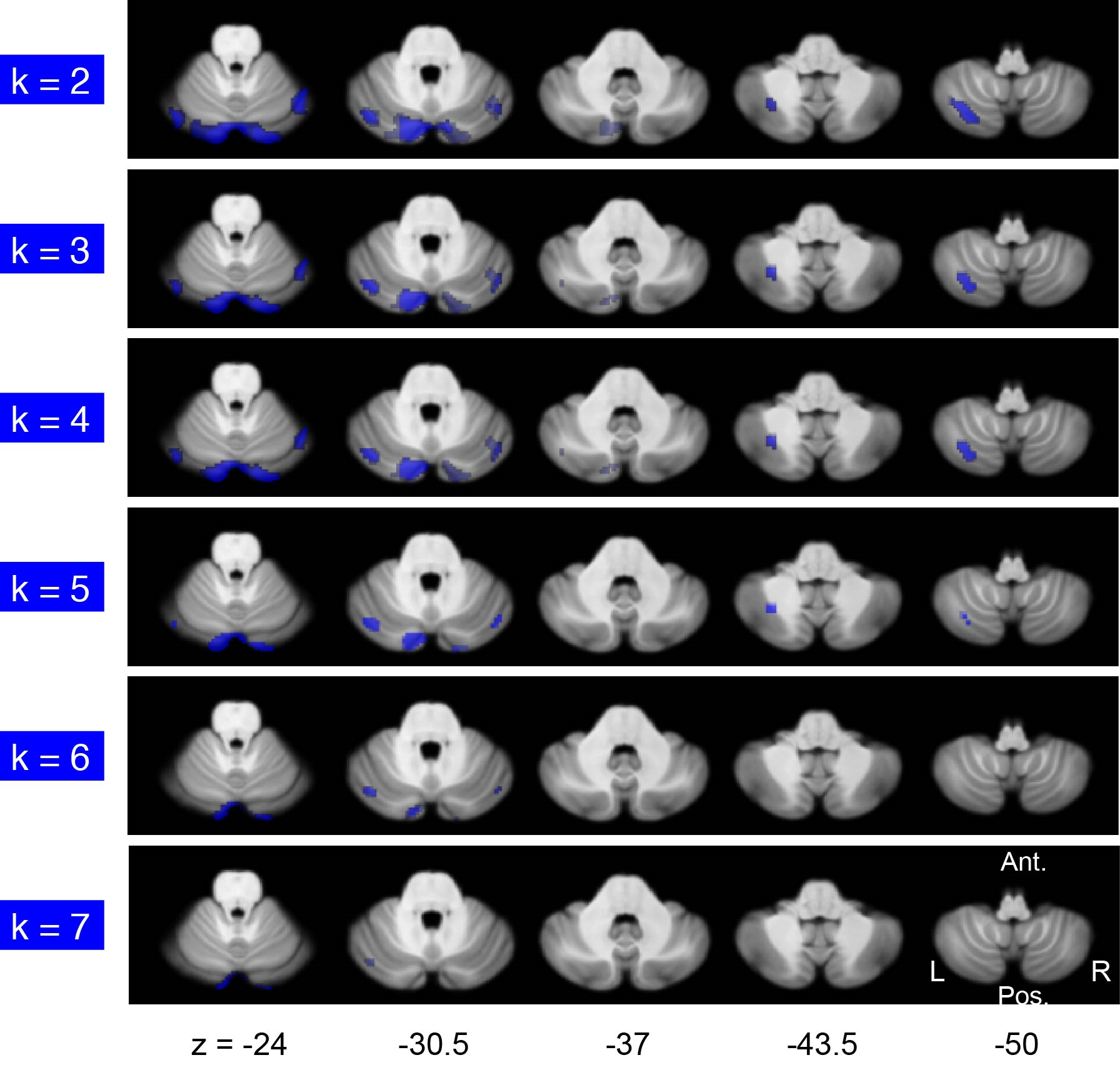

Supplement: S1 Fig — Highest correlations were found in the posterior region of the cerebellum for faster time constants. (TIF) [file pbio.1002312.s007.tif]

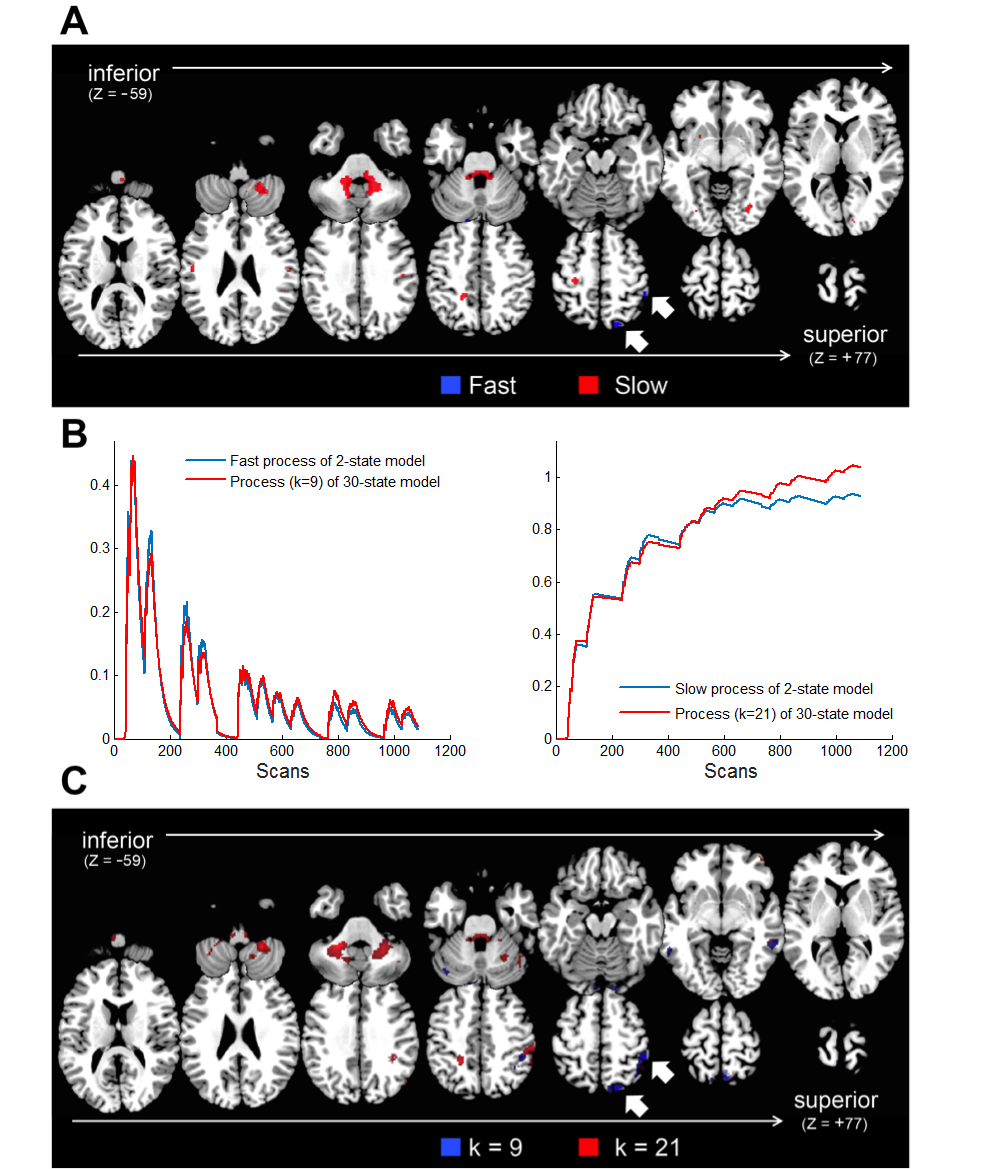

Supplement: S2 Fig — (A) We fitted the behavioral data with a two-state model and obtained the fast and slow process with time constants of 47.9 s and 1.5 h, respectively. We conducted a regression analysis of brain activity using the fast and the slow processes. The regression model included the four regressors that corresponded to fast and slow components for Tasks 1 and 2 and other regressors modeling effects of no interest such as hand movements, error, and reaction time. Results were thresholded at a lenient statistical level (p < 0.01 uncorrected) for each task, and regions that overlapped between the two tasks are indicated by colors (blue for the fast and red for the slow component). (B) The time constant of the fast (47.9 s; blue curve in the left panel) and the slow (1.5 h; blue curve in the right panel) states are very close to those from the suggested 30-state model, k = 9 (55.2 s; red curve in the left panel) and k = 21 (2.22 h; red curve in the right panel)—see S1 Table; the correlation coefficients between corresponding states in the two models are respectively 0.993 and 0.991. (C) For comparison, we thresholded results for the two time-constants (k = 9 and k = 21) from the 30-state model at p < 0.01 (uncorrected) for each task and indicated the regions overlapped between Tasks 1 and 2. Results are similar to those of the two-state model. (TIF) [file pbio.1002312.s008.tif]

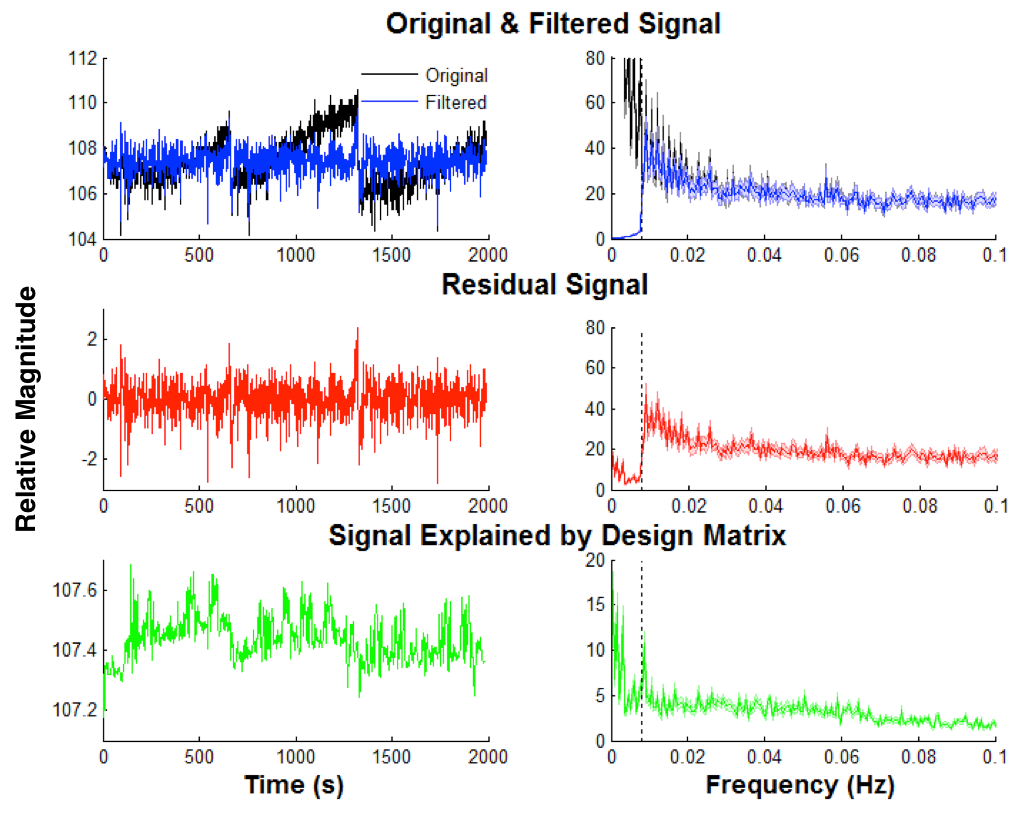

Supplement: S3 Fig — Here, we illustrate how the low-frequency components in the cerebellum were attenuated by the high-pass filter but remained sufficiently large to be correlated with the regressors with the slower time constants. Left panels show a representative BOLD signal in the right cerebellum (MNI: [22 −46 40]) for one subject (not averaged). Right panels show the mean and the SEM of frequency components (the line and the shaded region, respectively) across subjects for the same voxel. The top panels show the BOLD signal and its frequency components before and after high-pass filtering (using spm_filter.m with cutoff frequency: 0.0078 Hz, period: 128 s). As expected, the frequency components below the cut-off frequency (black dotted line) were attenuated; however, they were not completely eliminated. The middle panels show the residual signal from the BOLD signal and its frequency components not explained by a design matrix for the slow regressor (k = 30). The bottom panels show the signal explained by the design matrix and its frequency components. As can be seen on the bottom-right panel, the magnitude of the signal at low frequencies is still sufficiently large, resulting in significant correlation of the BOLD signals in the cerebellum with the slow regressor. (TIF) [file pbio.1002312.s009.tif]

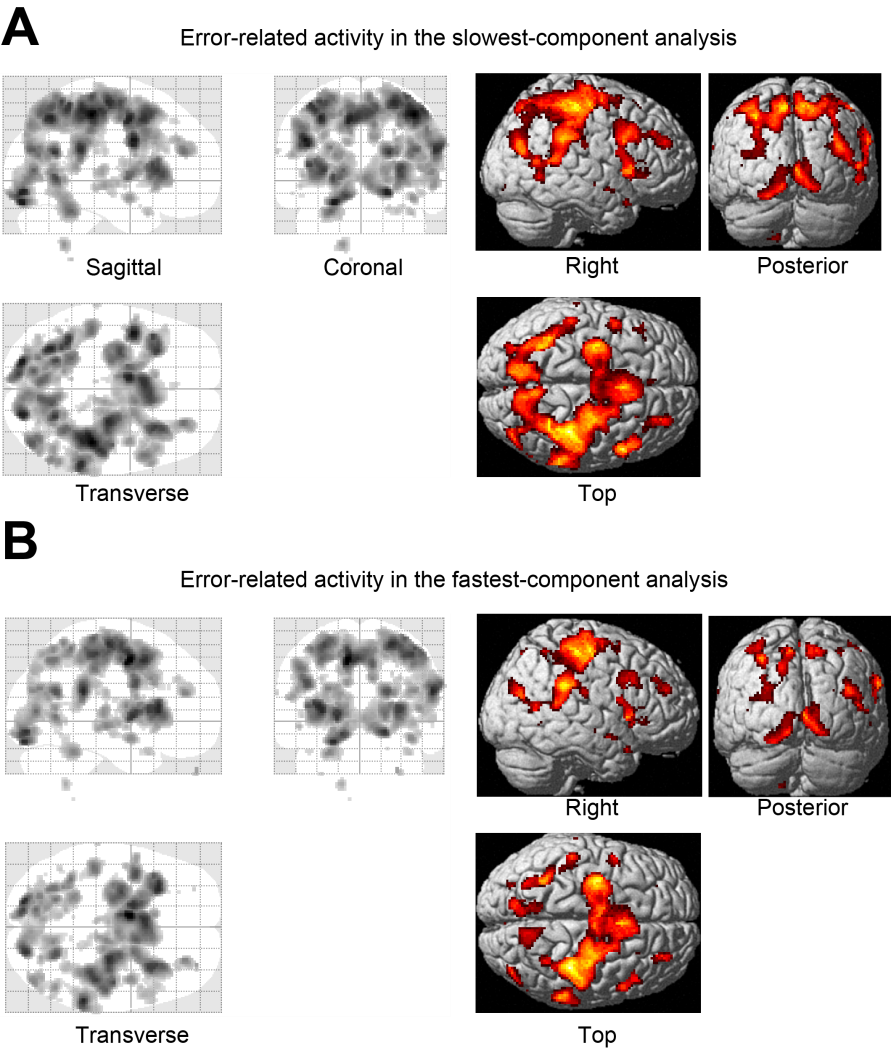

Supplement: S4 Fig — (A) Activity derived from the regression analysis of the slowest component. (B) Activity derived from the analysis of the fastest component. Activity was thresholded at p < 0.001 uncorrected for multiple comparisons. The left figures represent activity projected to the sagittal, coronal, and transverse planes (glass brain). The right figures show activity projected to the surface of the brain from the left, posterior, and top viewpoints. (TIF) [file pbio.1002312.s010.tif]

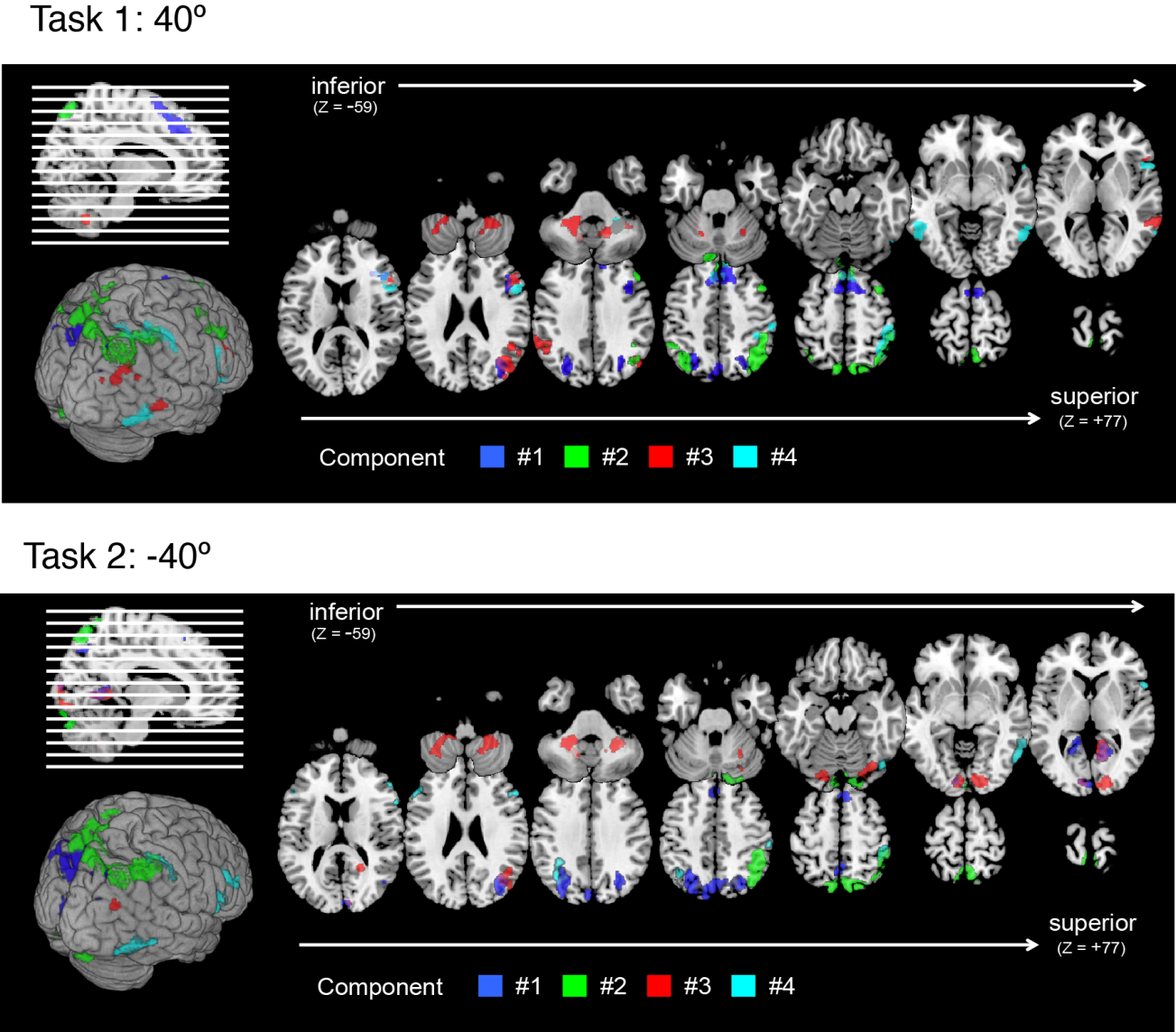

Supplement: S5 Fig — As described in the main text, the correlated brain activities were characterized with the top four components, two relatively fast components (first and second), an intermediate component (third), and one slow component (fourth). A similar pattern was found for Tasks 1 and 2. (TIF) [file pbio.1002312.s011.tif]

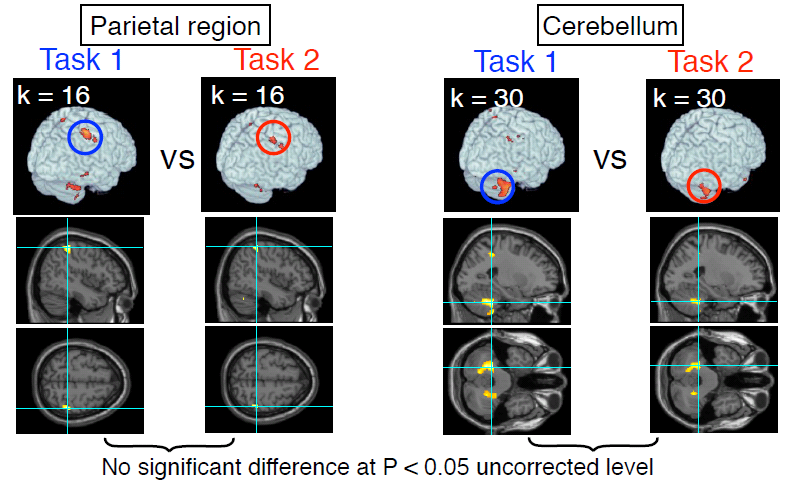

Supplement: S6 Fig — The correlated brain activities showed no significant difference between Tasks 1 and 2 in either the parietal (k = 16, τ k = 16.7 min) or the cerebellar (k = 30, τ k = 92.6 min) regions. (TIF) [file pbio.1002312.s012.tif]
