# Supplementary material for: Neural Substrates Related to Motor Memory with Multiple Timescales in Sensorimotor Adaptation
Source: PLoS Biol. 2015 Dec 8;13(12):e1002312. doi: 10.1371/journal.pbio.1002312 (PMC4672877; doi:10.1371/journal.pbio.1002312)
Supplement: S1 Table — (DOCX) [file pbio.1002312.s013.docx]

| ***k*** | **Time constant (*τ_k_* )** | ***k*** | **Time constant (*τ_k_* )** | ***k*** | **Time constant (*τ_k_* )** |
| --- | --- | --- | --- | --- | --- |
|  | | | | | |
| 1 | 2.00 s | 11 | 2.11 minutes | 21 | 2.22 hours |
| 2 | 3.03 s | 12 | 3.19 minutes | 22 | 3.36 hours |
| 3 | 4.58 s | 13 | 4.83 minutes | 23 | 5.08 hours |
| 4 | 6.94 s | 14 | 7.31 minutes | 24 | 7.69 hours |
| 5 | 10.50 s | 15 | 11.06 minutes | 25 | 11.65 hours |
| 6 | 15.90 s | 16 | 16.74 minutes | 26 | 17.63 hours |
| 7 | 24.07 s | 17 | 25.35 minutes | 27 | 26.69 hours |
| 8 | 36.43 s | 18 | 38.37 minutes | 28 | 40.41 hours |
| 9 | 55.15 s | 19 | 58.08 minutes | 29 | 61.17 hours |
| 10 | 83.48 s | 20 | 87.92 minutes | 30 | 92.59 hours |
