# Supplementary material for: Neural Substrates Related to Motor Memory with Multiple Timescales in Sensorimotor Adaptation
Source: PLoS Biol. 2015 Dec 8;13(12):e1002312. doi: 10.1371/journal.pbio.1002312 (PMC4672877; doi:10.1371/journal.pbio.1002312)
Supplement: S2 Table — (DOCX) [file pbio.1002312.s014.docx]

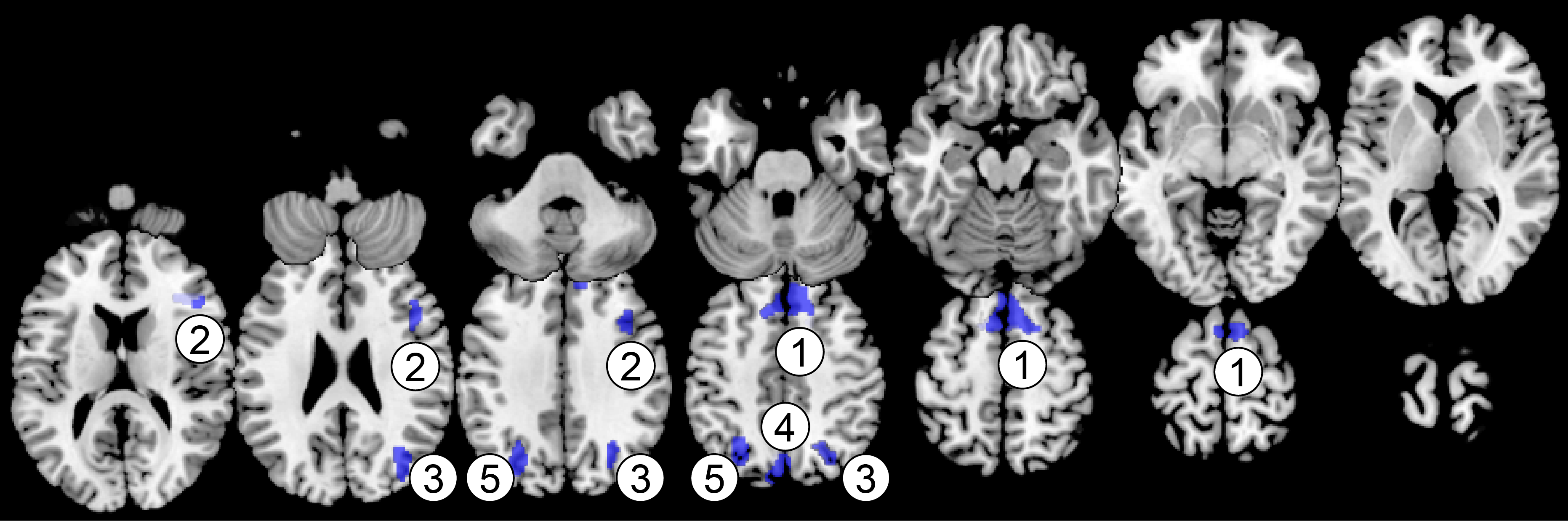


| Size | Cluster composition | | Peak coordinates | | | Eigen-value at peak |
| --- | --- | --- | --- | --- | --- | --- |
|  | Anatomical region | % | *x* | *y* | *z* |  |
| **(1) Supplementary Motor Area/Superior Frontal Gyrus (SMA/SFG)** | | | | | | |
| 955 | R Supplementary Motor Area *  L Supplementary Motor Area  R Superior Frontal Gyrus (medial)  L Superior Frontal Gyrus (medial) | 32.26  26.07  21.36  12.77 | -2 | 12 | 54 | 0.020063 |
|  |  |  |  |  |  |  |
| **(2) R Inferior Frontal Gyrus** | |  |  |  |  |  |
| 204 | R Inferior Frontal Gyrus  (Triangular part) *  R Inferior Frontal Gyrus  (Opercular part) | 40.20  37.75 | 40 | 16 | 28 | 0.017857 |
|  |  |  |  |  |  |  |
| **(3) R Medial Occipito-Parietal regions (MOP)** | | | | | | |
| 124 | R Middle Occipital Gyrus *  R Superior Occipital Gyrus | 45.82  34.66 | 36 | -76 | 20 | 0.016834 |
|  |  |  |  |  |  |  |
| **(4) L Precuneus** | |  |  |  |  |  |
| 119 | L Precuneus | 81.51 | -4 | -74 | 46 | 0.014213 |
|  |  |  |  |  |  |  |
| **(5) L Medial Occipito-Parietal regions (MOP)** | | |  |  |  |  |
| 268 | L Middle Occipital Gyrus  L Superior Parietal Gyrus *  L Inferior Parietal Gyrus | 37.69  30.22  23.13 | -28 | -64 | 48 | 0.014153 |

***Note***: Cluster size is represented by voxels (8 mm^3^/voxel). Clusters with more-than 100 voxels are listed in the order of eigen-value at the peak. Anatomical regions having more-than 10% of the volume of each cluster are listed. Asterisks indicate regions in which the peak exists. Shaded rows indicate clusters that were also found in the 1-st component of Task 2 (see Table S6).
