# Supplementary material for: Neural Substrates Related to Motor Memory with Multiple Timescales in Sensorimotor Adaptation
Source: PLoS Biol. 2015 Dec 8;13(12):e1002312. doi: 10.1371/journal.pbio.1002312 (PMC4672877; doi:10.1371/journal.pbio.1002312)
Supplement: S3 Table — (DOCX) [file pbio.1002312.s015.docx]

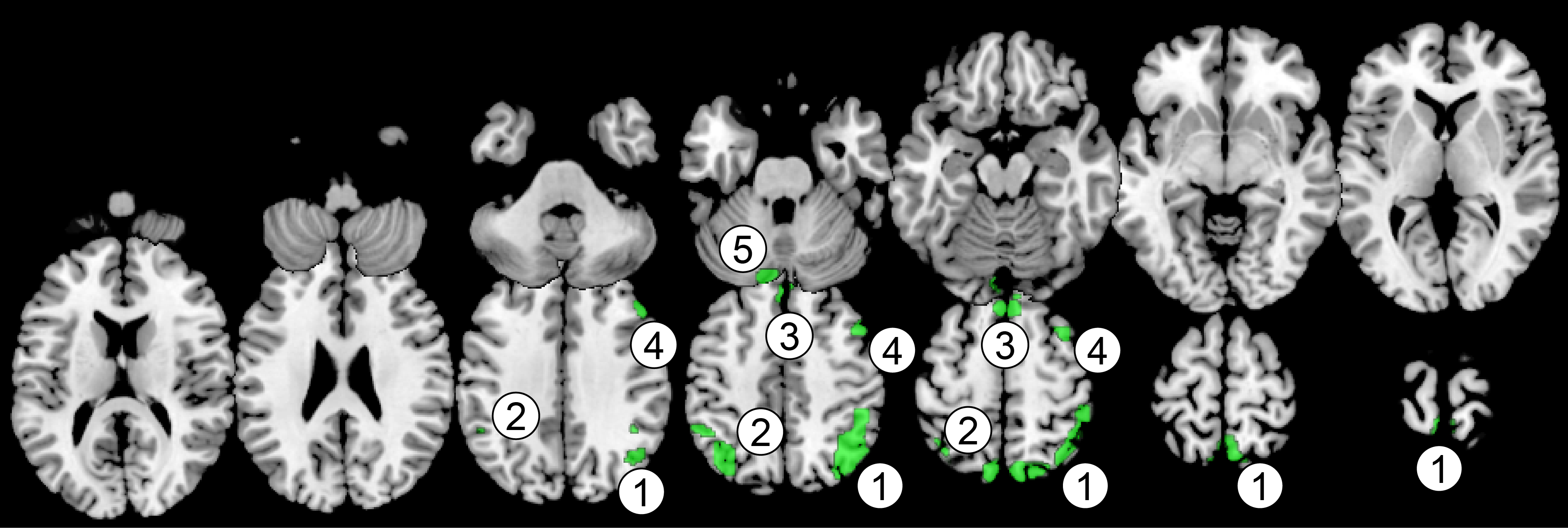


| Size | Cluster composition | | Peak coordinates | | | Eigen-value at peak |
| --- | --- | --- | --- | --- | --- | --- |
|  | Anatomical region | % | *x* | *y* | *z* |  |
| **(1) R Posterior part of Intraparietal Sulcus (pIPS)** | | | | | | |
| 1642 | R Angular Gyrus | 24.67 |  |  |  |  |
|  | R Inferior Parietal Gyrus | 20.89 |  |  |  |  |
|  | R Precuneus * | 15.90 | 2 | -58 | 72 | 0.018257 |
|  | R Superior Parietal Gyrus | 15.47 |  |  |  |  |
|  | L Precuneus | 15.23 |  |  |  |  |
|  |  |  |  |  |  |  |
| **(2) L Posterior part of Intraparietal Sulcus (IPS)** | | | | | | |
| 376 | L Inferior Parietal Gyrus | 60.37 |  |  |  |  |
|  | L Angular Gyrus * | 28.99 | -38 | -66 | 46 | 0.015667 |
|  | L Superior Parietal Gyrus | 10.11 |  |  |  |  |
|  |  |  |  |  |  |  |
| **(3) Supplementary Motor Area/Superior Frontal Gyrus (SMA/SFG)** | | | | | | |
| 238 | L Superior Frontal Gyrus (medial) | 47.48 |  |  |  |  |
|  | R Superior Frontal Gyrus (medial)* | 34.45 | 4 | 26 | 58 | 0.014029 |
|  | L Supplementary Motor Area | 10.08 |  |  |  |  |
|  |  |  |  |  |  |  |
| **(4) R Middle Frontal Gyrus (MFG)** | |  |  |  |  |  |
| 144 | R Middle Frontal Gyrus * | 100.00 | 48 | 26 | 36 | 0.011538 |
|  |  |  |  |  |  |  |
| **(5) Posterior Cerebellum (pCBL)** | |  |  |  |  |  |
| 141 | L Cerebellum Crus1 * | 40.43 | -2 | -88 | -16 | 0.011335 |
|  | L Cerebellum Crus2 | 34.75 |  |  |  |  |

***Note***: Conventions follow Table S2. Shaded rows indicate clusters that were also found in the 2-nd component of Task 2 (see Table S7).
