# Supplementary material for: Neural Substrates Related to Motor Memory with Multiple Timescales in Sensorimotor Adaptation
Source: PLoS Biol. 2015 Dec 8;13(12):e1002312. doi: 10.1371/journal.pbio.1002312 (PMC4672877; doi:10.1371/journal.pbio.1002312)
Supplement: S4 Table — (DOCX) [file pbio.1002312.s016.docx]

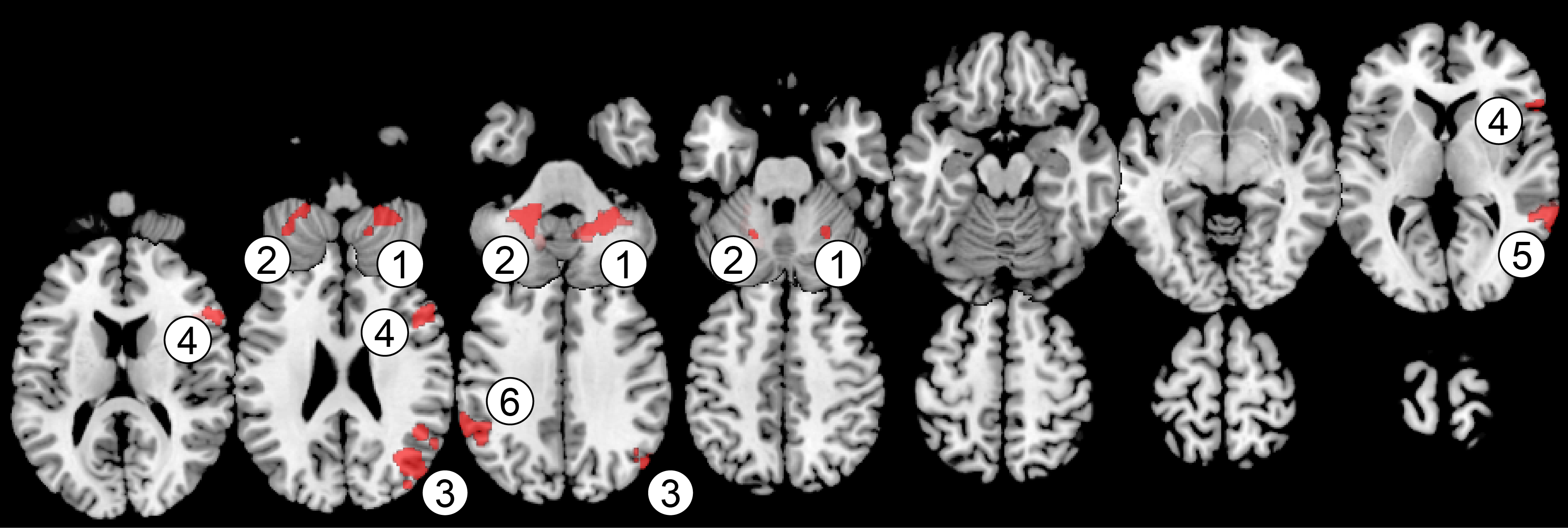


| Size | Cluster composition | | Peak coordinates | | | Eigen-value at peak |
| --- | --- | --- | --- | --- | --- | --- |
|  | Anatomical region | % | *x* | *y* | *z* |  |
| **(1) R Anterior-madeial Cerebellum (a-mCBL)** | | | | | | |
| 418 | R Cerebellum 8 * | 42.82 | 20 | -50 | -50 | 0.030590 |
|  | R Cerebellum 9 | 17.22 |  |  |  |  |
|  |  |  |  |  |  |  |
| **(2) L Anterior-madeial Cerebellum (a-mCBL)** | | | | | | |
| 415 | L Cerebellum 8 * | 20.00 | -16 | -46 | -38 | 0.026526 |
|  | L Cerebellum 6 | 10.36 |  |  |  |  |
|  |  |  |  |  |  |  |
| **(3) R Temporo-parietal Junction (TPJ)** | |  |  |  |  |  |
| 443 | R Angular Gyrus | 38.37 |  |  |  |  |
|  | R Middle Occipital Gyrus * | 37.92 | 30 | -68 | 22 | 0.023832 |
|  |  |  |  |  |  |  |
| **(4) R Inferior Frontal Gyrus** | |  |  |  |  |  |
| 261 | R Inferior Frontal Gyrus  (Triangular part) * |  | 58 | 24 | 14 | 0.017644 |
|  |  |  |  |  |  |  |
| **(5) R Middle Temporal Gyrus** | |  |  |  |  |  |
| 142 | R Middle Temporal Gyrus * | 99.30 | 52 | -46 | 10 | 0.016932 |
|  |  |  |  |  |  |  |
| **(6) L Supramarginal Gyrus** | |  |  |  |  |  |
| 132 | L Supramarginal Gyrus * | 63.64 | -50 | -50 | 30 | 0.016347 |
|  | L Angular Gyrus | 25.76 |  |  |  |  |
|  | L Inferior Parietal Gyrus | 10.61 |  |  |  |  |

***Note***: Conventions follow Table S2. Shaded rows indicate clusters that were also found in the 3-rd component of Task 2 (see Table S8).
