# Supplementary material for: Neural Substrates Related to Motor Memory with Multiple Timescales in Sensorimotor Adaptation
Source: PLoS Biol. 2015 Dec 8;13(12):e1002312. doi: 10.1371/journal.pbio.1002312 (PMC4672877; doi:10.1371/journal.pbio.1002312)
Supplement: S5 Table — (DOCX) [file pbio.1002312.s017.docx]

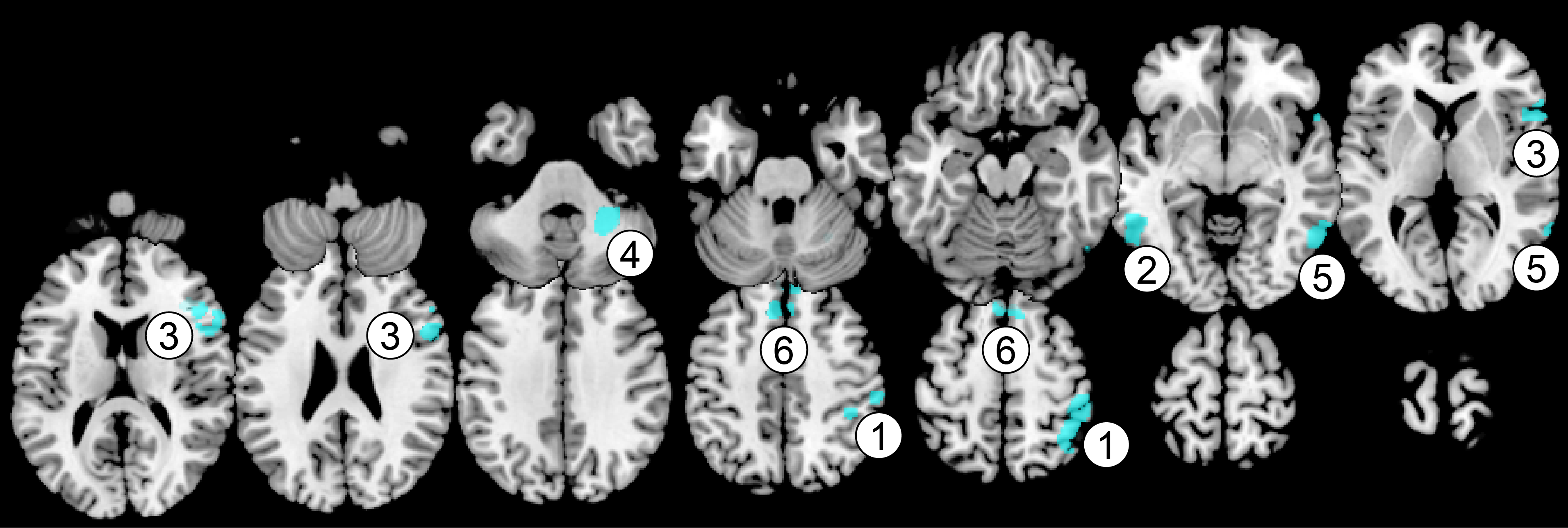


| Size | Cluster composition | | Peak coordinates | | | Eigen-value at peak |
| --- | --- | --- | --- | --- | --- | --- |
|  | Anatomical region | % | *x* | *y* | *z* |  |
| **(1) R Anterior part of Intraparietal Sulcus (aPIS)** | | | | | | |
| 405 | R Postcentral Gyrus | 39.01 |  |  |  |  |
|  | R Inferior Parietal Gyrus * | 32.35 | 46 | -36 | 58 | 0.042429 |
|  | R Superior Parietal Gyrus | 23.21 |  |  |  |  |
|  |  |  |  |  |  |  |
| **(2) L Middle/Inferior Temporal regions (M/ITG)** | | | | | | |
| 109 | L Middle Temporal Gyrus * | 52.29 | -60 | -60 | -6 | 0.033785 |
|  | L Inferior Temporal Gyrus | 47.71 |  |  |  |  |
|  |  |  |  |  |  |  |
| **(3) R Inferior Frontal Gyrus (IFG)** | |  |  |  |  |  |
| 446 | R Inferior Frontal Gyrus  (Opercular part) * | 56.50 | 58 | 14 | 4 | 0.023449 |
|  | R Inferior Frontal Gyrus  (Triangular part) | 35.20 |  |  |  |  |
|  |  |  |  |  |  |  |
| **(4) R Anterior-medial Cerebellum (a-mCBL)** | | | | | | |
| 146 | R Cerebellum 6 * | 14.38 | 24 | -46 | -40 | 0.021860 |
|  |  |  |  |  |  |  |
| **(5) R Middle/Inferior Temporal Gyri (M/ITG)** | | |  |  |  |  |
| 148 | R Inferior Temporal Gyrus * | 75.68 | 54 | -66 | -10 | 0.019900 |
|  | R Middle Temporal Gyrus | 19.59 |  |  |  |  |
|  |  |  |  |  |  |  |
| **(6) Superior Frontal Gyrus (SFG)** | |  |  |  |  |  |
| 231 | L Superior Frontal Gyrus (medial)* | 49.78 | -4 | 26 | 52 | 0.017223 |
|  | R Superior Frontal Gyrus (medial) | 30.74 |  |  |  |  |
|  | L Supplementary Motor Area | 12.55 |  |  |  |  |

***Note***: Conventions follow Table S2. Shaded rows indicate clusters that were also found in the 4-th component of Task 2 (see Table S9).
