# Supplementary material for: Neural Substrates Related to Motor Memory with Multiple Timescales in Sensorimotor Adaptation
Source: PLoS Biol. 2015 Dec 8;13(12):e1002312. doi: 10.1371/journal.pbio.1002312 (PMC4672877; doi:10.1371/journal.pbio.1002312)
Supplement: S6 Table — (DOCX) [file pbio.1002312.s018.docx]

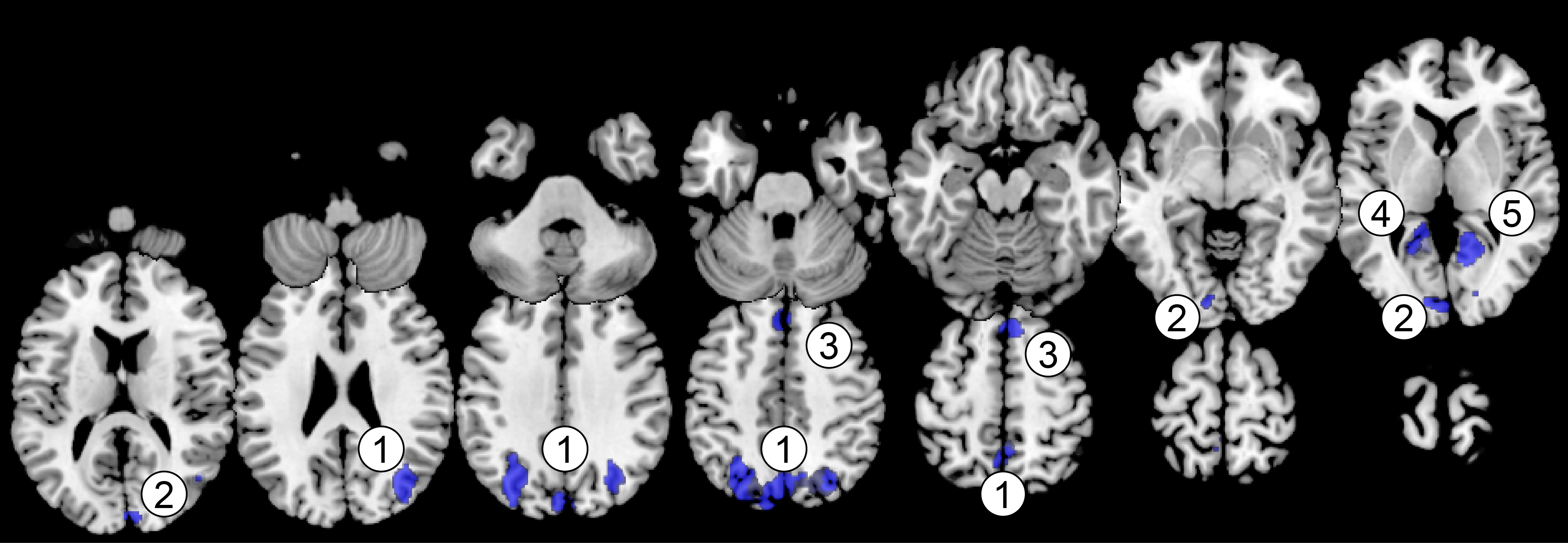


| Size | Cluster composition | | Peak coordinates | | | Eigen-value at peak |
| --- | --- | --- | --- | --- | --- | --- |
|  | Anatomical region | % | *x* | *y* | *z* |  |
| **(1) Medial Occipito-Parietal regions (MOP)** | | |  |  |  |  |
| 1775 | L Precuneus | 18.54 |  |  |  |  |
|  | L Middle Occipital Gyrus | 12.85 |  |  |  |  |
|  | R Middle Occipital Gyrus | 12.68 |  |  |  |  |
|  | L Superior Parietal Gyrus | 12.62 |  |  |  |  |
|  | L Cuneus * | 5.41† | -2 | -80 | 32 | 0.02157 |
|  |  |  |  |  |  |  |
| **(2) Calcarine Fissure** | |  |  |  |  |  |
| 251 | L Calcarine Fissure | 62.15 |  |  |  |  |
|  | R Calcarine Fissure | 18.33 |  |  |  |  |
|  | R Cuneus * | 8.37† | 8 | -90 | 16 | 0.01980 |
|  |  |  |  |  |  |  |
| **(3) Supplementary Motor Area/Superior Frontal Gyrus (SMA/SFG)** | | | | | | |
| 246 | L Superior Frontal Gyrus (medial) | 36.59 | 0 | 30 | 52 | 0.018628 |
|  | L Supplementary Motor Area | 33.33 |  |  |  |  |
|  | R Supplementary Motor Area | 18.70 |  |  |  |  |
|  | R Superior Frontal Gyrus (medial) | 11.38 |  |  |  |  |
|  |  |  |  |  |  |  |
| **(4) L Lingual Gyrus** | |  |  |  |  |  |
| 133 | L Lingual Gyrus | 56.39 |  |  |  |  |
|  | L Precuneus | 25.56 |  |  |  |  |
|  | L Calcarine Fissure * | 8.27† | -22 | -58 | 4 | 0.015416 |
|  |  |  |  |  |  |  |
| **(5) R Lingual Gyrus** | |  |  |  |  |  |
| 215 | R Lingual Gyrus * | 63.26 | 12 | -64 | 2 | 0.014826 |
|  | R Calcarine Fissure | 28.37 |  |  |  |  |

***Note***: Conventions follow Table S2 except that an anatomical region having less-than 10% of each cluster volume is listed if the peak exists in that region (†). Shaded rows indicate clusters that were also found in the 1st component of Task 1 (see Table S2).
