# Supplementary material for: Neural Substrates Related to Motor Memory with Multiple Timescales in Sensorimotor Adaptation
Source: PLoS Biol. 2015 Dec 8;13(12):e1002312. doi: 10.1371/journal.pbio.1002312 (PMC4672877; doi:10.1371/journal.pbio.1002312)
Supplement: S7 Table — (DOCX) [file pbio.1002312.s019.docx]

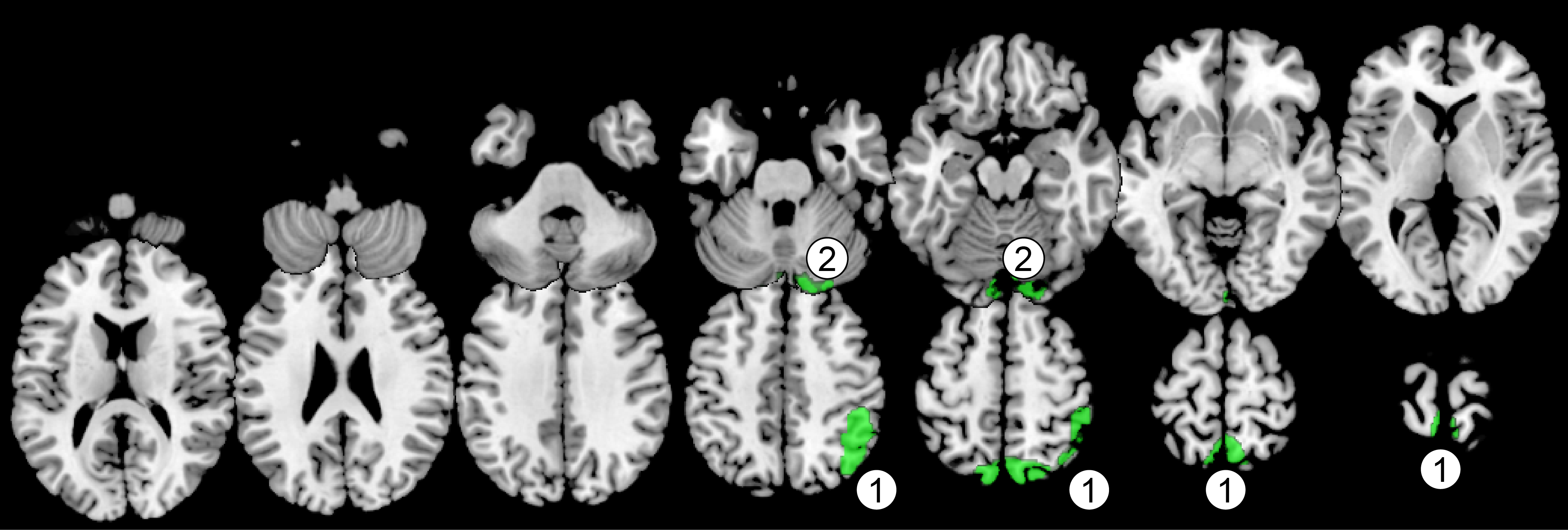


| Size | Cluster composition | | Peak coordinates | | | Eigen-value at peak |
| --- | --- | --- | --- | --- | --- | --- |
|  | Anatomical region | % | *x* | *y* | *z* |  |
| **(1) R Posterior part of Intraparietal Sulcus (pIPS)** | | | | | | |
| 1810 | Parietal_Inf_R | 26.63 |  |  |  |  |
|  | Precuneus_R | 19.61 | 8 | -72 | 60 | 0.018263 |
|  | Precuneus_L | 17.85 |  |  |  |  |
|  | Angular_R | 13.43 |  |  |  |  |
|  | Parietal_Sup_R | 13.09 |  |  |  |  |
|  |  |  |  |  |  |  |
| **(2) Posterior Cerebellum (pCBL)** | |  |  |  |  |  |
| 391 | R Cerebellum_Crus1 | 39.13 | 16 | -90 | -20 | 0.014103 |
|  | L Cerebellum_Crus1 | 13.81 |  |  |  |  |
|  | L Calcarine Fissure | 10.49 |  |  |  |  |

***Note***: Conventions follow Table S2. Shaded rows indicate clusters that were also found in the 2-nd component of Task 1 (see Table S3).
