# Supplementary material for: Neural Substrates Related to Motor Memory with Multiple Timescales in Sensorimotor Adaptation
Source: PLoS Biol. 2015 Dec 8;13(12):e1002312. doi: 10.1371/journal.pbio.1002312 (PMC4672877; doi:10.1371/journal.pbio.1002312)
Supplement: S8 Table — (DOCX) [file pbio.1002312.s020.docx]

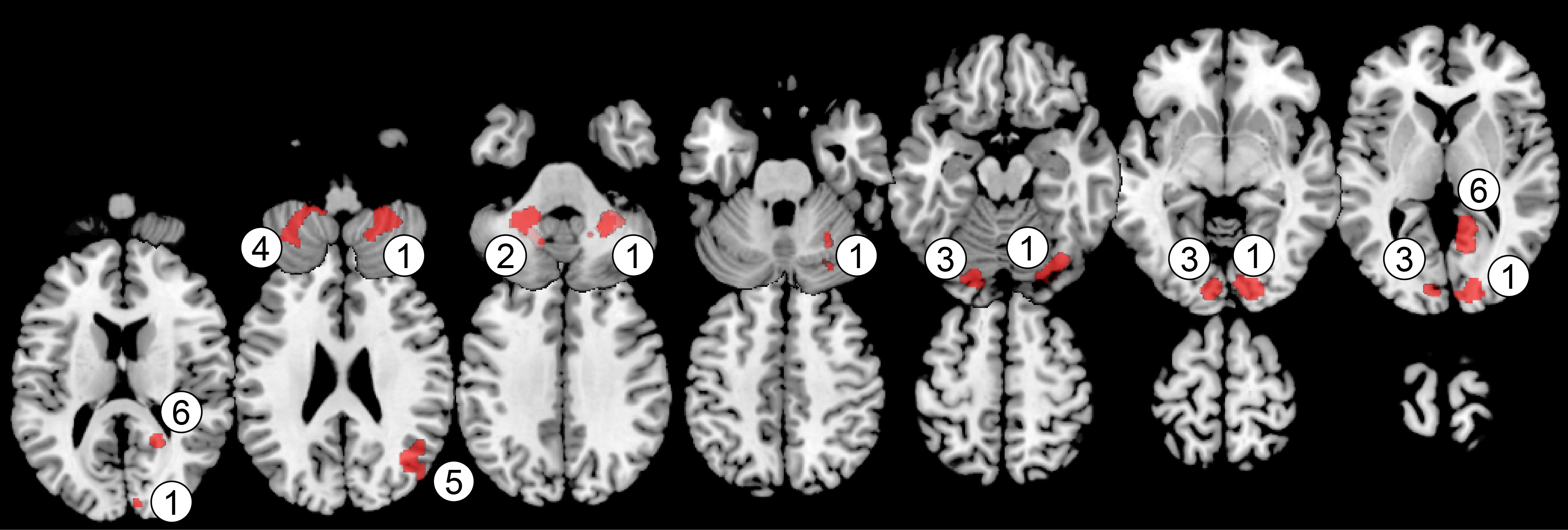


| Size | Cluster composition | | Peak coordinates | | | Eigen-value at peak |
| --- | --- | --- | --- | --- | --- | --- |
|  | Anatomical region | % | *x* | *y* | *z* |  |
| **(1) R Anterior-medial Cerebellum (a-mCBL)** | | | | | | |
| 826 | R Cerebellum 8 * | 24.94 | 20 | -54 | -50 | 0.033339 |
|  | R Calcarine Fissure | 16.22 |  |  |  |  |
|  | R Lingual Gyrus | 16.10 |  |  |  |  |
|  | R Cerebellum 6 | 11.26 |  |  |  |  |
|  |  |  |  |  |  |  |
| **(2) L Anterior-medial Cerebellum (a-mCBL)** | | | | | | |
| 187 | L Cerebellum 6 | 94.12 | -16 | -46 | -38 | 0.026016 |
|  |  |  |  |  |  |  |
| **(3) L Calcarine Fissure** | |  |  |  |  |  |
| 173 | L Calcarine Fissure | 58.38 |  |  |  |  |
|  | L Lingual Gyrus | 26.01 |  |  |  |  |
|  | L Middle Occipital Gyrus * | 0.58† | -14 | -90 | -6 | 0.020974 |
|  |  |  |  |  |  |  |
| **(4) L Anterior-medial Cerebellum (a-mCBL)** | | | | | | |
| 174 | L Cerebellum 8 * | 84.48 | -34 | -52 | -48 | 0.018924 |
|  | L Cerebellum 9 | 12.07 |  |  |  |  |
|  |  |  |  |  |  |  |
| **(5) R Temporo-parietal Junction (TPJ)** | |  |  |  |  |  |
| 134 | R Angular Gyrus | 44.78 |  |  |  |  |
|  | R Middle Occipital Gyrus | 38.81 |  |  |  |  |
|  | R Middle Temporal Gyrus * | 2.99† | 38 | -66 | 22 | 0.015810 |
|  |  |  |  |  |  |  |
| **(6) R Lingual Gyrus** | |  |  |  |  |  |
| 263 | R Lingual Gyrus * | 59.32 | 12 | -64 | 2 | 0.014844 |
|  | R Calcarine Fissure | 22.81 |  |  |  |  |
|  | R Precuneus | 14.45 |  |  |  |  |

***Note***: Conventions follow Table S2 except that an anatomical region having less-than 10% of each cluster volume is listed if the peak exists in that region (†). Shaded rows indicate clusters that were also found in the 3-rd component of Task 1 (see Table S4).
