# Supplementary material for: Neural Substrates Related to Motor Memory with Multiple Timescales in Sensorimotor Adaptation
Source: PLoS Biol. 2015 Dec 8;13(12):e1002312. doi: 10.1371/journal.pbio.1002312 (PMC4672877; doi:10.1371/journal.pbio.1002312)
Supplement: S9 Table — (DOCX) [file pbio.1002312.s021.docx]

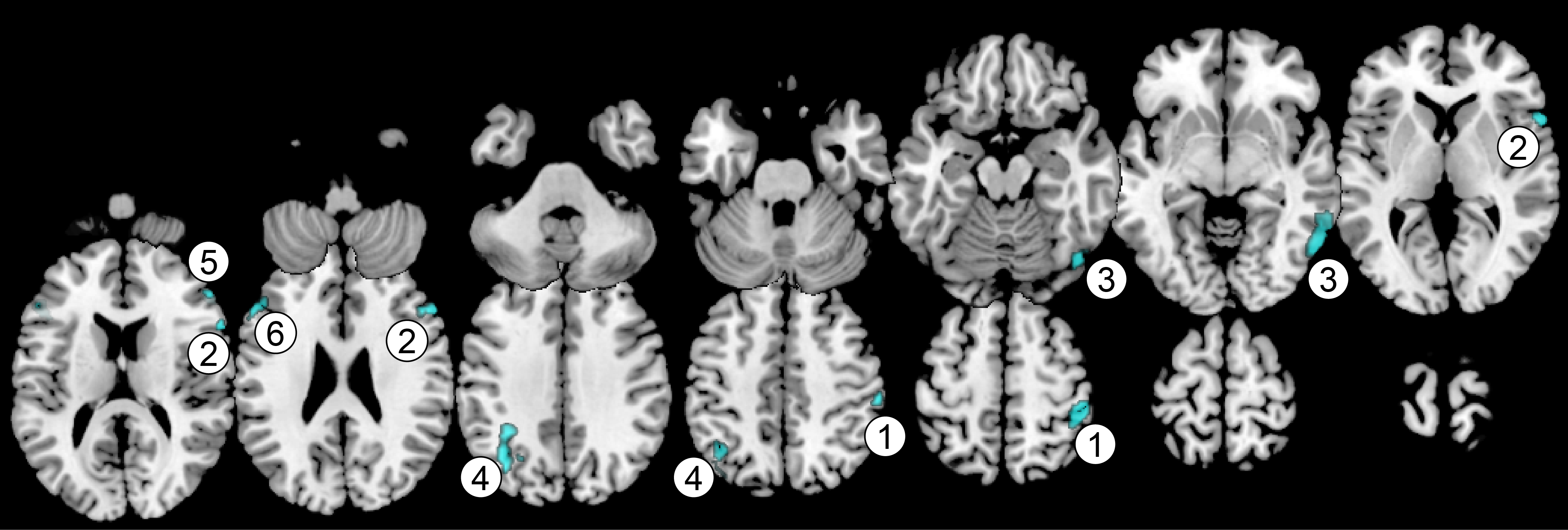


| Size | Cluster composition | | Peak coordinates | | | Eigen-value at peak |
| --- | --- | --- | --- | --- | --- | --- |
|  | Anatomical region | % | *x* | *y* | *z* |  |
| **(1) R Anterior part of Intraparietal Sulcus (aIPS)** | | | | | | |
| 185 | R Postcentral Gyrus * | 53.51 | 46 | -36 | 60 | 0.027464 |
|  | R Inferior Parietal Gyrus | 28.65 |  |  |  |  |
|  | R Supramarginal Gyrus | 10.81 |  |  |  |  |
|  |  |  |  |  |  |  |
| **(2) R Inferior Frontal Gyrus (IFG)** | |  |  |  |  |  |
| 109 | R Inferior Frontal Gyrus  (Opercular part) * | 80.73 | 62 | 16 | 12 | 0.026097 |
|  | R Inferior Frontal Gyrus  (Triangular part) | 11.93 |  |  |  |  |
|  |  |  |  |  |  |  |
| **(3) R Middle/Inferior Temporal Gyri (M/ITG)** | | |  |  |  |  |
| 273 | R Inferior Temporal Gyrus * | 52.75 | 54 | -66 | -12 | 0.025043 |
|  | R Middle Temporal Gyrus | 27.11 |  |  |  |  |
|  | R Inferior Occipital Gyrus | 14.65 |  |  |  |  |
|  |  |  |  |  |  |  |
| **(4) L Temporo-parietal Junction (TPJ)** | |  |  |  |  |  |
| 287 | L Angular Gyrus | 46.69 |  |  |  |  |
|  | L Inferior Parietal Gyrus | 27.53 |  |  |  |  |
|  | L Middle Occipital Gyrus * | 15.33 | -36 | -66 | 32 | 0.018302 |
|  |  |  |  |  |  |  |
| **(5) R Inferior Frontal Gyrus (IFG)** | |  |  |  |  |  |
| 121 | R Inferior Frontal Gyrus  (Triangular part) | 92.56 | 54 | 28 | 18 | 0.017389 |
|  |  |  |  |  |  |  |
| **(6) L Inferior Frontal Gyrus (IFG)** | |  |  |  |  |  |
| 134 | L Inferior Frontal Gyrus  (Triangular part) | 97.01 | -56 | 26 | 18 | 0.016658 |

***Note***: Conventions follow Table S2. Shaded rows indicate clusters that were also found in the 4-th component of Task 1 (see Table S5).
