# Supplementary material for: Neural Substrates Related to Motor Memory with Multiple Timescales in Sensorimotor Adaptation
Source: PLoS Biol. 2015 Dec 8;13(12):e1002312. doi: 10.1371/journal.pbio.1002312 (PMC4672877; doi:10.1371/journal.pbio.1002312)
Supplement: S1 Text — (DOC) [file pbio.1002312.s022.doc]

The multiple adaptation model in the main text contains no interference between tasks. In a previous model with two time constants, we have shown that, interference between tasks was prominent in the faster process [1, 2], and others have similarly shown interferences in adaptation. Accordingly, such interference can be seen for instance in the first trial of the first block of Task 2 in Figure 2A. However, it can be observed in the figure that the interference seems minimal in following blocks. This is not surprising because the potential for interference was reduced in our experimental design in which switching between the tasks occurred only between blocks. We therefore assumed here a simple model without interference between states.

We validated this assumption of no interference by comparing the proposed model to an alternative model with an interference parameter, γ in the contextual cue as $\mathbf{c}=(1\text{ γ})^{T}$for Task 1 and $\mathbf{c}=(\text{γ 1})^{T}$for Task 2. To compare the models, we generated 10,000 bootstrapped data sets (sampling with repetition) and calculated the Bayesian Information Criterion of the proposed and the alternative models. We then performed bootstrap *t*-test to validate the proposed model at the 5-percent significance level [3]. There was no significant difference in the Bayesian Information Criterion between the two models (Bootstrap *t*-test p = 0.1297). In addition, the estimated (95% confidence interval) interference parameter was close to zero, 0.0746 (0-0.311), supporting the proposed model without interference.

**References**

1. Lee JY, Schweighofer N. Dual Adaptation Supports a Parallel Architecture of Motor Memory. J Neurosci. 2009;29(33):10396-404. doi: Doi 10.1523/Jneurosci.1294-09.2009. PubMed PMID: WOS:000269087300023.

2. Smith MA, Ghazizadeh A, Shadmehr R. Interacting adaptive processes with different timescales underlie short-term motor learning. PLoS Biol. 2006;4(6):e179.

3. DiCiccio TJ, Efron B. Bootstrap confidence intervals. Statistical science. 1996:189-212.
