# Supplementary material for: Neural Substrates Related to Motor Memory with Multiple Timescales in Sensorimotor Adaptation
Source: PLoS Biol. 2015 Dec 8;13(12):e1002312. doi: 10.1371/journal.pbio.1002312 (PMC4672877; doi:10.1371/journal.pbio.1002312)
Supplement: S2 Text — (DOC) [file pbio.1002312.s023.doc]

A possible concern with our exploratory regression analysis is that activity is not related to memory state but to errors. This is an especially valid concern for the faster states, because the memory states of the fast components correlate with the errors used for updating adaptation, with high error values at the initial stages and low error values at the late stages of adaptation (see main text). Here, we therefore analyzed the activity significantly correlated with the error-related regressors (*p* < 0.001 uncorrected) in the regression analysis of the slowest component (*k* = 30), which is unlikely positively correlated with the error. Activity was found in many regions including the parietal regions and the cerebellum (S3A Fig), which are consistent with previous studies on performance error [1, 2]. Then we checked the error-related activity in the analysis of the fastest component, and found a similar activity pattern (S3B Fig). Thus, although these results do not completely exclude possibility that the activity correlated with fast components is involved in error processing, they suggest that the error-associated nuisance regressors appropriately explained away the error in the regression analyses.

**References**

1. Diedrichsen J, Hashambhoy Y, Rane T, Shadmehr R. Neural correlates of reach errors. J Neurosci. 2005;25(43):9919-31. Epub 2005/10/28. doi: 25/43/9919 [pii]

10.1523/JNEUROSCI.1874-05.2005. PubMed PMID: 16251440.

2. Schlerf J, Ivry RB, Diedrichsen J. Encoding of sensory prediction errors in the human cerebellum. J Neurosci. 2012;32(14):4913-22.
