# Supplementary material for: Neural Substrates Related to Motor Memory with Multiple Timescales in Sensorimotor Adaptation
Source: PLoS Biol. 2015 Dec 8;13(12):e1002312. doi: 10.1371/journal.pbio.1002312 (PMC4672877; doi:10.1371/journal.pbio.1002312)
Supplement: S3 Text — (DOC) [file pbio.1002312.s024.doc]

The increased behavioral performance due to adaptation (i.e., decrease in endpoint directional errors) may cause decrease in variance of low-level movement kinematics, which could then contribute to the better classification accuracy of the two rotational conditions in our MVPA analysis. We first compared the variance of the directional errors for each session. We found significant difference of variance in errors from the 1^st^ and 2^nd^ sessions (*F*_(20, 20)_ = 3.33, *p* *<* 0.01 [*p <* 0.02 corrected for two comparisons with the Bonferroni method]), but no significant difference between the 2^nd^ and 3^rd^ sessions (*F*_(20, 20)_ = 0.90, *p* = 0.82). Similarly, the mean of errors showed no significant difference between the 2^nd^ and 3^rd^ sessions (*t*_(20)_ = 1.05, *p* = 0.31) despite of significant difference across sessions (*F*_(2, 60)_ = 10.07, *p <* 0.001). In addition, we found no significant correlation between the difference in classification accuracy of the cerebellum and that of the variability (S.D.) of absolute directional errors between 2^nd^ and 3^rd^ session for individual subjects (correlation coefficient = -0.27, *p* = 0.24). Finally, reaction times and movement times showed no significant difference across sessions (*F*_(2, 60)_ = 0.0582, *p* = 0.944, *F*_(2, 60)_ = 1.71, *p* = 0.189). In sum, these results show that performance already plateaued in the 2^nd^ session, and increased classification accuracy in the cerebellum is unlikely to be caused by behavioral confounds**.**
